# Multifactorial Basis and Therapeutic Strategies in Metabolism-Related Diseases

**DOI:** 10.3390/nu13082830

**Published:** 2021-08-18

**Authors:** João V. S. Guerra, Marieli M. G. Dias, Anna J. V. C. Brilhante, Maiara F. Terra, Marta García-Arévalo, Ana Carolina M. Figueira

**Affiliations:** 1 Brazilian Center for Research in Energy and Materials (CNPEM), Brazilian Biosciences National Laboratory (LNBio), Polo II de Alta Tecnologia—R. Giuseppe Máximo Scolfaro, Campinas 13083-100, Brazil; joao.guerra@lnbio.cnpem.br (J.V.S.G.); marieli.dias@lnbio.cnpem.br (M.M.G.D.); maiara.terra@lnbio.cnpem.br (M.F.T.); 2 Graduate Program in Pharmaceutical Sciences, Faculty Pharmaceutical Sciences, University of Campinas, Campinas 13083-970, Brazil; 3 Graduate Program in Functional and Molecular Biology, Institute of Biology, State University of Campinas (Unicamp), Campinas 13083-970, Brazil; anna.brilhante@lnbr.cnpem.br; 4 Brazilian Center for Research in Energy and Materials (CNPEM), Brazilian Biorenewables National Laboratory (LNBR), Polo II de Alta Tecnologia—R. Giuseppe Máximo Scolfaro, Campinas 13083-100, Brazil

**Keywords:** NCDs, obesity, diabetes, MAFLD, cardiovascular diseases, metabolism

## Abstract

Throughout the 20th and 21st centuries, the incidence of non-communicable diseases (NCDs), also known as chronic diseases, has been increasing worldwide. Changes in dietary and physical activity patterns, along with genetic conditions, are the main factors that modulate the metabolism of individuals, leading to the development of NCDs. Obesity, diabetes, metabolic associated fatty liver disease (MAFLD), and cardiovascular diseases (CVDs) are classified in this group of chronic diseases. Therefore, understanding the underlying molecular mechanisms of these diseases leads us to develop more accurate and effective treatments to reduce or mitigate their prevalence in the population. Given the global relevance of NCDs and ongoing research progress, this article reviews the current understanding about NCDs and their related risk factors, with a focus on obesity, diabetes, MAFLD, and CVDs, summarizing the knowledge about their pathophysiology and highlighting the currently available and emerging therapeutic strategies, especially pharmacological interventions. All of these diseases play an important role in the contamination by the SARS-CoV-2 virus, as well as in the progression and severity of the symptoms of the coronavirus disease 2019 (COVID-19). Therefore, we briefly explore the relationship between NCDs and COVID-19.

## 1. Introduction

Non-communicable diseases (NCDs), also known as chronic diseases, are not directly transmissible from one person to another, and are the combination of genetic, physiological, environmental, and behavioral factors. The main NCDs are diabetes, cardiovascular diseases, cancers, and chronic respiratory diseases. The first three are associated with metabolic changes that increase the risk of suffering them. These changes are hypertension, overweight/obesity, hyperglycemia, and hyperlipidemia (WHO). The importance of these diseases was highlighted in the report of the World Health Organization (WHO) [1], in which it was reported that over 50% of the 57 million deaths worldwide, in 2016, occurred from diabetes (1.6 million people), cancer (9 million), and cardiovascular diseases (17.9 million) [2], posing a significant global health challenge. As well as genetics, unhealthy habits such as smoking, harmful use of alcohol, physical inactivity, and a calorie-rich diet are determinants for developing metabolism-related diseases; such behavioral factors lead to metabolic disorders such as hypertension, hyperglycemia, hyperlipidemia, and obesity, which comprise the major NCDs risk factors [3]. Furthermore, over the past two decades, the understanding of the association between metabolic disorders and metabolic associated fatty liver disease (MAFLD)—a less commonly discussed NCD—has placed it as an emerging risk factor for diabetes, cancer, and cardiovascular diseases (CVDs) [4].

In view of NCDs’ global threat, the WHO has adopted priority targets to reduce NCDs mortality and risk factor prevalence until 2025 [1]. However, the global prevalence of risk factors is still concerning. In 2015, one in four men and one in five women had hypertension, corresponding to 22% of the adults aged 18 years and over [5]. In recent decades, hypertension prevalence in high-income countries has declined; on the other hand, many low- and middle-income countries had stable or increasing levels. The contrast among income groups was slight regarding blood glucose levels in 2014. Most countries had between 7% and 9% of the population with hyperglycemia—except for the Eastern Mediterranean Region, which showed the highest levels (14%) [6]. Globally, the adult obesity prevalence in 2016 was 13% (650 million people); it is almost three times higher than in 1975 [5]. Although adult obesity rates distinguish between low- (7% of the population) and high-income countries (25%), the numbers keep rising in all income groups [2]. The prevalence of childhood obesity has also increased at higher rates in recent decades. From 1975 to 2016, the number of obese children and adolescents worldwide increased approximately eight-fold, reaching 124 million in 2016 [5]. Among obese and diabetic individuals, about 70–80% have MAFLD; this is the leading chronic liver disease worldwide, with a prevalence of 20–30%, affecting 1.8 billion people [7].

Countries’ ability to deal with NCDs proved to be even more critical during the coronavirus disease 2019 (COVID-19) pandemic, since the association between NCDs and COVID-19 severity have been reported. Hypertension, ischemic heart disease, type 2 diabetes (T2D), and cancer were among the most prevalent NCDs in Italian COVID-19 victims [8]. This association has also been observed in Spain, China, and the USA [9,10,11]. Additionally, a Chinese study showed that severe patients and non-survivors were overweight or obese, suggesting an association between body mass index (BMI) and COVID-19 severity [12]. In this scenario, given COVID-19′s restrictive measures, economic instability, and health crisis, NCDs’ prevention and management became even more challenging [13].

Following the multifactorial nature of metabolism-related diseases, their prevention and treatment consist of multidisciplinary strategies to tackle the physiological and metabolic impairments. Lifestyle interventions are the primary recommendations; however, some cases also require surgical or pharmacotherapeutic approaches [14]. Although some NCD medicines are well established—such as metformin and insulin for diabetes [15] and antihypertensive agents to control some CVDs [16]—so far, no agent has been approved for MAFLD [17]. Furthermore, the discovery of additional metabolic mechanisms of NCDs pathogenesis stimulates the search for new metabolic modulators. Since pharmacotherapy’s efficacy and safety rely on the agent’s mechanism of action, drug design and development are constantly advancing. In addition to traditional combination therapies, this field advances towards the evaluation of multitarget ligands and emerging therapeutic strategies [18].

Considering the global relevance of NCDs and the constant research progress, this article reviews the current understanding about NCDs and their related risk factors, with a focus on obesity, diabetes, MAFLD, and CVDs, summarizing the knowledge about their pathophysiology and highlighting available and emerging therapeutic strategies. In addition, we briefly discuss the relationship between these conditions and their related risk factors and COVID-19. A better understanding of this critical health issue and potential therapeutic approaches can help mitigate NCDs’ global impact.

## 2. Obesity

Obesity is a multifactorial and preventable disease, defined as an excessive accumulation of body fat [19]. In recent decades, obesity has been a major global health issue, with a considerable impact on morbidity, mortality, and healthcare expenditure [20,21]. This issue has increased rapidly, reaching epidemic proportions. About 39% of the world’s adult population is overweight and, among this, 13% was obese in 2016. In 2017, more than 4 million deaths worldwide were due to obesity and its associated comorbidities [1]. This epidemic includes childhood obesity, that has also raised dramatically in recent years [5]. Thus, it increases the risk of early-onset chronic consequences, such as elevated blood pressure, CVDs occurrence, and impaired glucose metabolism, that usually evolves to T2D [22,23,24]. Moreover, childhood obesity increases the risk of obesity in adults more than five-fold compared to non-obese children [25]. In addition, morbidity and mortality are also elevated later in life [26].

Obesity is essentially a long-term imbalance between energy consumption and expenditure, which creates an oversupply of energy, resulting in excess fat storage. The complexity of this pathogenesis relies on its multiple causes, such as environmental, sociocultural, physiological, genetic, epigenetic, and various other factors that act together to contribute to the origin, as well as the persistence, of this condition. In the last century, the world’s social and economic changes favored a positive energy balance. The industrialization process allows the population to increase the consumption of energy-rich and often highly-palatable foods, but poor in nutrients [20]. At the same time, this process of urbanization decreases the levels of daily physical activity and increases a sedentary lifestyle [27,28].

Beyond the global factors, our individual socioeconomic and cultural environment also affects the obesity incidence. Together with abundant tasty food and low physical activity, contemporary elements, such as medications with weight gain side effects, reduced sleep time, endocrine disruptors, and epigenetic effects are components that favor the obesity epidemic [29]. Hereditary factors also play a role in this condition. In this sphere, genetics, family history, and ethnic/racial variants can increase the susceptibility to obesity. The variability of population predisposition is predicted to range from 40% to 70% due to genetic differences [30,31]. There are more than 100 genes identified as obesity-related at different contribution scales. The fat mass and obesity-associated (FTO) gene is known to predispose obesity through an effect on BMI [32,33]. Another harmful variant would be defective leptin receptor or leptin production and abnormalities in the proopiomelanocortin (POMC) gene [34,35].

Genes work together with the environment in a complex network that combines metabolic processes and body weight adjustment to regulate energy balance [36,37]. Feeding behavior is regulated by neurons that are excited or suppressed by neuropeptide hormones that act as signals for food intake and energy expenditure. Among them, ghrelin is an orexigenic hormone secreted in the gastrointestinal (GI) tract in a fasted state, involving hunger perceptions [38]. Moreover, hunger is associated with food palatability, such as visual, olfactory, emotional eating, and increased reward-responses to food stimuli [39].

In short-term energy regulation, nutrient-derived signals from GI tract adjust appetite through amino acids, gut-brain peptides, and various neurotransmitters. Food intake induces a reduction in circulating ghrelin levels while increasing secretion of the anorectic hormones cholecystokinin (CCK), peptide YY3-36 (PYY), glucagon-like peptide-1 (GLP-1), and oxyntomodulin [40]. Long-term energy balance involves several central and peripheral mechanisms that act in a finely tuned regulation network to maintain metabolic homeostasis. Insulin and leptin secretion signal feedback information in response to food intake, regulating, in addition the appetite, the thermogenesis process, fat deposition, and cognitive processes involved in food consumption [36,39].

Excess adiposity causes alterations in whole-body homeostasis, leading to functional impairments in various metabolic functions [41] and considerably increases the risk of metabolic diseases. The pathophysiology of obesity culminates in distinct homeostatic mechanisms that hinder weight loss and benefit further weight gain. The storage of energy excess leads to an increase in the number (hyperplasia) and size (hypertrophy) of the adipocytes, as well as the ectopic distribution of lipid deposits in regions, such as blood vessels, visceral fat, cardiac fat, and muscles, in a process called dyslipidemia [42]. The enlargement of fat cells increases the number of pro-inflammatory factors, including leptin, interleukin-6 (IL-6), monocyte chemotactic protein 1 (MCP-1), and lipid metabolism metabolites, such as lactate and free fatty acids (FFA). Simultaneously, they lessen the release of adiponectin, an adipokine related to insulin sensitivity, and interleukin-10 (IL-10), an anti-inflammatory cytokine [43]. Together, adipocyte products can affect the brain and peripheral nervous system, modifying metabolism, and inflammatory processes.

Hypertrophic adipocytes work together with the microbiome to increase the inflammatory environment [44]. The gut microbiota is an essential environmental factor in energy balance, acting directly in food digestion to increase energy absorption. This process produces metabolites, such as lipopolysaccharides (LPS), short-chain fatty acids (FAs), and secondary bile acids, which act as signaling molecules, modulating hunger, nutrient absorption, gut motility, and energy balance [44]. In obesity, microbiota imbalance induced by high energy dense diet increase microbial products, such as LPS, that activate innate immunity, contributing to low-grade inflammation via increased expression of inflammatory mediators (e.g., TLR family, NOD-like receptor family and cytokines) and macrophage infiltration [45].

Increased circulating FFA and adipokines cause peripheral-tissue and nervous system dysfunction. Leptin is one of the multiple factors excessively secreted by hypertrophic fat cells. This adipokine acts directly on lipid accumulation by inhibiting hunger, signaling the cessation of adipocyte fat storage [46]. Plasma leptin levels are positively correlated with adiposity. With an abundance of food, secretion of leptin suppresses energy intake, while stimulating energy expenditure. However, in obesity, a prolonged increase in plasma leptin levels leads to decreased detection of the peripheral energy status, which culminates in ineffective satiety detection despite high energy storage and leptin levels [46,47]. This damaged mechanism leads to gradual weight gains due to a continuous positive energy balance, feeding the continuous cycle of hunger.

Excessive food intake, lipotoxicity, and elevated lipid accumulation induce the expression of cytokines and activation of cells involved in innate immunity [48]. As obesity progresses, adipose tissue macrophage infiltration increases in number and changes the gene expression profile to a greater inflammatory environment [49,50]. The increased inflammatory response includes proinflammatory M1 macrophages shift, NK cells activation, interferon γ (INF-γ) and chemokines production, accumulation of CD8+ T-cells and TH1-polarized lymphocytes [50,51,52].

Metabolic inflammation caused by circulating FFA also induces alterations in insulin release. Obesity and overweight are the main predictors to T2D development, a metabolic disease that relies on defective insulin signaling. Insulin sensitivity, as well as insulin secretion, can be reduced by obesity influence. The chronic abundance of energy maintains constantly high levels of plasma glucose, which lessen the β-cells response to incretins, decreasing insulin sensitivity and leading to insulin resistance (IR), a process that is also mediated by tumor necrosis factor α (TNFα), IL-1β, extracellular signal-regulated protein kinases 1 and 2 (ERK1/2), and c-Jun N-terminal kinases (JNKs) signaling [53,54,55]. At the same time, factors such as lipotoxicity, incretin resistance and glucotoxicity decrease β-cell mass, which, in turn, decreases insulin secretion. This impaired insulin signaling and lipotoxicity are also crucial factors to the development of MAFLD [56].

Many other diseases are also associated with obesity. The dyslipidemia process can induce CVDs, such as hypertension, myocardial infarction, and stroke [57,58]. The mechanical stress caused by over-weight leads to musculoskeletal disorders, such as osteoarthritis, as well as sleep apnea [59,60]. Increased levels of tumorigenic molecules, such as insulin-like growth factor 1 (IGF-1), are associated with several types of cancer, e.g., mammary, ovarian, prostate, gastrointestinal, liver, and renal cancer [61,62]. However, some obese patients do not have associated risk factors, a phenomenon described as ‘healthy obese’ [63]. Obesity is also a major cause of Alzheimer’s disease [64,65], decreased life expectancy [66,67], reduced quality of life, lower productivity, social disadvantages, and early retirement [67,68]. Furthermore, obesity is closely related to some mental illnesses, such as clinical depression [69], anxiety [70], and other brain disorders [71,72].

### 2.1. Management of Obesity

Obesity management demands a multidisciplinary approach with individualized programs. The development of a management strategy may consider the factors that contribute to obesity, as well as the overweight degree, the pre-existence of one or more associated diseases, and individual limitations. Currently, interventions are mainly based on controlling food intake and energy expenditure with changes in dietary and physical activity. The goal of treatment is the initial loss of at least 5% of the patient’s total weight. The greater the initial weight loss leads to better and faster health recovery. However, in some cases, behavioral changes alone are not enough, so pharmacotherapeutic or surgical interventions can also be part of the treatment [73].

#### 2.1.1. Lifestyle Interventions

Comprehensive lifestyle intervention is the cornerstone of obesity management, and adjunctive treatment may be required for individuals with more compromised health, or for those who do not achieve the required weight loss [74]. Assisted behavioral changes help patients to understand and monitor their feeding behavior, creating a more conscious lifestyle [75]. However, despite the essential role of these programs in initial lifestyle changes, they fail in long-term attendance. The initial recommendations alone are not enough for re-education, without psychological assistance and adequate physical training, initial adherence to lifestyle changes is often abandoned, thus weight regain is frequent after the end of re-education programs [76].

The so-called “westernization” of lifestyle in recent decades facilitates the increase in drivers of obesity. Technologies are progressively evolving to make our lives more comfortable, as a result, the general population tends to be less active, increasing the odds of consuming more than expending. Automations and computer-based work are the majority of occupations, thus lowering the daily expenditure. This Western lifestyle also contributes to an increase in convenience foods (e.g., frozen, canned, and pre-cooked), greater fast-food availability, more effective food marketing, and larger food portions, which also corresponds with consumption of fewer home cooked meals. These factors contribute to an obesogenic environment that leads to rising levels of obesity worldwide [77,78].

Dietary interventions are essential for weight loss. Different dietary approaches with caloric restriction provide this effect, maintaining a negative energy balance. Usually, the guidelines recommend a 30% restriction on daily energy consumption, which is equivalent to 300 to 500 Kcal per day, added to an improvement in the nutritional quality of foods [79]. The choice of the calorie-restrict diet should be individualized according to the patient’s condition (i.e., gender, age, physical activity status) and preferences, in order to maximize program adherence [80].

Typically, different diets have variations in macronutrient composition; however, these differences do not imply a more effective approach. The key to effective weight loss is the long-term patient adherence to the diet, so it is important that diet choice can be matched to individual preferences [81].

Several dietary strategies can be used to induce weight loss by prioritizing one of several healthy dietary patterns. However, many of them are not nutritionally advisable or not properly considered healthy. Among those considered nutritionally recommended are dietary approach to stop hypertension (DASH), metabolic syndrome reduction in navarra (RESMENA), and Mediterranean diets, combined with low-fat (Fat: 10%–19%) and low-carbohydrate (Carbohydrate: 20%) content. These diets are commonly prescribed for weight loss and are equally effective with patient commitment [82].

DASH is an eating plan with positive effects on weight control and the cardiovascular system [83]. In this diet, the priority should be a high intake of fruits, vegetables, whole grains, and nuts. Fat-free and low-fat dairy products, as well as fish, poultry, and some legumes, such as beans, are also included. Vegetable oils are allowed, but tropical oils (such as coconut and palm oils), sugar-sweetened, refined sugar, and foods rich in saturated fats should be restricted [80].

The Mediterranean diet also emphasizes plant-based foods, such as fruits and vegetables, whole grains, legumes, and nuts. However, it also includes high intake of olive oil, moderate intake of fish and poultry, red wine in moderation, and low intake of red meat and sweets [80]. It is a very popular diet due to its role in lowering the risk of developing obesity, T2D, and CVDs. This diet also has positive effects during pregnancy, with a lower risk of fetal deficiency and promoting fetal development [84].

The RESMENA diet is a variant of the Mediterranean diet that reduces calorie intake by 30% and requires 30% of energy intake from protein. This diet also emphasizes consumption of anti-oxidant-rich fruits and vegetables and higher meal frequency (seven meals a day) [85]. In addition to weight loss, this diet reduces android fat mass (a region associated with hepatic steatosis) and waist circumference. Moreover, several biochemical parameters are improved, such as reduced transaminase levels, and maintenance of uric acid and serum glucose, indicating this diet as a good treatment option for obesity [85,86,87].

Intermittent fasting (IF) is another strategy that can induce weight loss. This eating plan has various arrangements within the premise of carrying out periods with little or no food consumption, interspersed with normal food intake on a recurrent basis [88]. During the ‘feeding window’, calorie intake is low and balanced, while during the ‘fasting window’, individuals ingest non-caloric drinks, such as water, coffee, and teas without any kind of sugar. [89]. In the most common IF, time-restricted feeding (TRF), the daily caloric intake must be consumed within a defined time window, followed by a fasting window that can range from 12 to 24 h [90]. Other protocols include fasting for up to 24 h twice a week and eating without restriction in the remaining days [91]; and alternate-day fasting (ADF), with no food restriction on eating days and no caloric intake on fasting days [91]. In addition to weight loss promoted by the caloric reduction that occurs naturally in IF, it promotes a metabolic shift that positively affects lipid and glucose metabolism, also improving diabetes, cardiovascular system (e.g., stroke), cancers, and neurological disorders, such as Alzheimer’s disease and Parkinson’s disease [88].

Another critical aspect is the inclusion of physical activity in the patient’s routine [92], which offers several benefits in addition to weight loss. Enhanced metabolic rates contribute to lowering the risk of CVDs and T2D. Additionally, the increase in muscle tissue improves bone health and joint stabilization. Moreover, physical activity promotes endorphin release and can contribute to overcome depression [93,94].

The initial recommendation is at least 150 min of moderate exercise or 75 min of vigorous physical activity per week [95]. Both aerobic and resistance training are recommended for weight loss. Although aerobic training improves cardiovascular function, resistance training promotes strength and muscle growth, which in turn increases the basal metabolic rate and, therefore, daily energy expenditure. The combination of both types of training demonstrated greater improvement in physical function and reduction in frailty compared to the isolated interventions [96].

Together, physical activity combined with dietary modification can promote optimal outcomes in overweight and obese patients when considering lifestyle interventions; however, in some cases, a pharmacological approach should be included [97].

#### 2.1.2. Pharmacotherapy

According to the Endocrine Society guidelines [70,73,76], weight loss drugs should be considered in cases of BMI >30 or BMI from 27 to 29 with at least one comorbidity. Still, pharmacotherapy should be prescribed as adjunctive therapy and does not exclude dietetic and physical activity improvements [98]. Obesity pharmacotherapy treatment reinforces dietary intervention that results in caloric deficit. Weight-loss medications act by decreasing appetite, helping to resist binge eating or decreasing caloric absorption. Appetite suppressants may act on leptin or anorexigenic pathways. The combination of these strategies improves the efficiency of initial lifestyle changes, as well as maintenance of lost weight.

Currently, there is no single optimal medication to treat the whole spectrum of obesity. The effectiveness of a particular medication is proven with the loss of at least 5% of total weight occurring after three months of treatment. Other efficiency criteria are the improvement in current comorbidities, prevention of new associated diseases, and maintenance of weight loss [74,76,98]. Still, weight loss must be realistic and aim for long-term adherence. In most cases, 5%–10% of total weight loss in six months is achievable and sustainable over the long term.

Orlistat is the only anti-obesity medication that acts directly on the GI tract, inhibiting long-chain FAs absorption by blocking pancreatic lipase action. It is a naturally occurring lipstatin derivative that acts by binding to and inhibiting pancreatic and gastric lipases. Inactivated enzymes are unable to hydrolyze the triglycerides (TG) of dietary fat to absorbable FFA, thus decreasing dietary fat absorption by 30% of the recommended therapeutic dose [73,99]. Orlistat side effects include GI problems, such as abdominal pain, fecal urgency, flatulence, and oily stool. However, these symptoms can be ameliorated by following a low-fat diet with no more than 30% of total calories from fat and with the addition of a fiber supplement. As Orlistat reduces the absorption of fat-soluble vitamins (A, D, E, and K), multivitamin supplementation is also recommended to guarantee adequate nutritional balance [99].

Phentermine is the most frequently prescribed anti-obesity drug. It is an adrenergic agonist that acts on the central nervous system (CNS) and increases norepinephrine release, reducing appetite, and increasing the basal metabolic rate [99,100]. Phentermine causes mild increase in heart rate and blood pressure. Therefore, its monotherapy is only approved for short-term use (three months) and in younger patients without coronary disease or hypertension history. However, this medication is contraindicated for patients suffering from insomnia and anxiety disorders [101,102].

Topiramate is a gamma-aminobutyric acid (GABA) receptor modulator initially approved for seizures and migraine treatment. Topiramate administration in epilepsy treatment promoted significant weight loss, persuading the interest in this drug for obesity treatment [103]. The mechanism of action of Topiramate on weight loss is not yet totally understood; however, it is known as an appetite suppressant and satiety enhancer, acting as a neurostabilizer and enhancing thermogenesis [104].

The association of phentermine/topiramate extended-release was the first combination drug approved by the US Food and Drug Administration (FDA) in 2012 for long-term obesity treatment [105,106]. The combination of these two drugs induces additive and dose-dependent weight loss by targeting different pathways at the same time, being, therefore, more effective than monotherapy with these medications. Weight loss induced by phentermine/topiramate extended-release use is associated with improvement in various comorbid risk factors, such as improved glycemic control, lower blood pressure and TGs, and increased high-density lipoprotein (HDL)-cholesterol, reducing also T2D progression, even in the reduced use of complementary medications [106]. This medication should not be prescribed to individuals with CVD or a history of anxiety or insomnia due to the phentermine component.

A naltrexone/bupropion combination controls appetite and improves energy utilization [107,108]. Naltrexone is an opioid antagonist prescribed for alcohol and opioid dependence [109,110]. Bupropion inhibits serotonin, dopamine, and norepinephrine reabsorption, which regulates central reward pathways triggered by food stimuli. Its monotherapy is approved as an antidepressant and smoking cessation treatment [111]. Collectively, they activate POMC neurons, promoting the release of alpha melanocyte-stimulating hormone (α-MSH), a neuropeptide involved in body energy regulation. At the same time, naltrexone is also important in antagonizing an inhibitory feedback loop that limits anorectic ability of bupropion [112,113].

Liraglutide is a glucagon-like peptide 1 (GLP-1) receptor agonist, which acts directly on satiety signals, delaying gastric emptying, leading to reduced food intake. This is the only anti-obesity drug administered in the form of subcutaneous injection. The peptide binds to the GLP-1 receptor augmenting insulin secretion. Insulin release increases glucose uptake, lowering the glucose plasma level. Liraglutide also retards gastric emptying and decreases appetite [114,115]. Simultaneous use of liraglutide with insulin/insulin secretagogues may increase the hypoglycemic risk. Liraglutide mechanism of action does not involve neurotransmitters, therefore, it is indicated for patients who are also taking psychiatric medications [114,115].

#### 2.1.3. Bariatric Surgery

Due to the high associated risks, bariatric surgery is recommended only in severe obesity, when BMI >40 or BMI >35 and there is at least one associated disease [116]. Currently, there are various types of intervention that result in different weight loss degrees. Each approach has different levels of associated benefits and risks that must be considered in conjunction with individual comorbidities and the patient’s history [117]. The three major surgical interventions used are: (I) Laparoscopic adjustable gastric band (LAGB)—the least invasive of the procedures, a band is placed around the stomach in a way that decreases in size; (II) Roux-en-Y gastric bypass (RYGB)—the removal of a large part of the stomach and the remaining portion is connected to the intestine, reducing the space available for food; (III) Laparoscopic sleeve gastrectomy (LSG), in which a large part of the stomach is also removed, but maintains the natural connection with the intestine [116,117].

#### 2.1.4. New Drugs and Strategies

Recent discoveries in the modulation of the complex system that underlies energy homeostasis and obesity pathways unveils new perspectives in obesity drug discovery. Leptin is a central target in energy homeostasis that acts as a nutrient sensor, interrupting hunger signals. Obese patients are usually leptin-resistant and have higher levels of leptin, so manipulating leptin signaling to induce its sensitivity is one of the strategies currently explored [118]. Metreleptin is a recombinant human leptin analogue used in lipodystrophic disorders treatment, lowering hepatic steatosis and improving insulin sensitivity, hyperglycemia, and hypertriglyceridemia [119,120]. Its use in obesity treatment has been considered to help normalize decreased leptin levels caused by weight loss [121].

Another class of leptin signal modulation is the use of leptin sensitizers. Pramlintide is a synthetic amylin analogue that acts on short-term satiety signaling, delaying gastric emptying, thus reducing food intake [122]. Davalintide is another amylin mimetic peptide that has a greater affinity to amylin, calcitonin and calcitonin gene-related peptide receptors, which causes enhanced pharmacological actions on satiety signals [123].

Semaglutide is a novel GLP-1 agonist with an extended half-life that allows subcutaneous administration once a week. This peptide also has increased affinity for GLP-1 receptor and demonstrates superior efficacy in weight loss when compared to liraglutide [124,125]. Oral GLP-1 agonists are being tested as alternatives to injectable agents. In addition to the semaglutide in oral form [126], TTP-054 and ZYOGI have demonstrated promising results in effective weight loss with minimal side effects [127].

ZP4165 is a gastric inhibitory peptide analogue that acts by inducing insulin release and decreases hemoglobin A1c (HbA1c) levels in animal studies. Its action also involves the GLP-1 pathway, enhancing GLP-1 induced weight loss, suggesting that administration in combination with GLP-1 analogues may be a promising treatment for obesity [128]. Another mechanism studied to control the GLP-1 pathway is the use of dual agonists, such as the oxyntomodulin, a peptide co-secreted with GLP1 L-cells, in response to nutritional stimuli. Oxyntomodulin is a glucagon receptor (GcgR)/GLP-1 receptor agonist that has been demonstrated to suppress appetite and increase energy expenditure, thereby decreasing food intake [129]. Nonetheless, it has only short-term effects, which leads to studies with synthetic dual agonists with increased half-life, such as MEDI0382 [130], and tirzepatide [131]. A triple agonist for GLP-1, glucagon and GIP receptors, the triagonist 1706 is also in trial phase, demonstrating effectiveness in weight loss [132].

Cannabinoid receptor type 1 (CB1) neutral antagonists stimulate anorexigenic signaling, leading to weight loss by reducing food intake [133]. AM-6545 is a novel peripheral CB1 antagonist that has limited penetration in CNS and has demonstrated promising effects on weight loss, without the central side effects of the formerly commercialized CB1 antagonist rimonabant. AM-6545 presented high affinity and selectivity for the CB1 receptor, with dose-dependent reduction in food intake and food-reinforced behavior [134,135].

Cetilistat is a novel lipase inhibitor, similar to orlistat. Cetilistat treatment has demonstrated significant weight loss and improvement in glycemic control and lipid profiles, with a lower potential for GI side effects, such as diarrhea, flatulence, and oily spotting attributed to orlistat [136].

The utilization of vaccines to prevent or treat is a novel therapeutic approach to obesity management. Anti-obesity vaccines use the immune response logic to restrain appetite-stimulating hormones and decrease nutrient absorption. Ghrelin, an orexigenic hormone secreted by stomach cells, is one of these anti-obesity strategies. Anti-ghrelin vaccine lessens food intake and orexigenic signals while increasing energy expenditure in pigs. However, in human clinical trials, this vaccine did not show an additional weight loss, even with a strong antibody response to ghrelin [137]. Another anti-obesity vaccine under development is the anti-somatostatin, which promises to remove the inhibitory effects of somatostatin on growth hormone (GH) and IGF-1 secretion, thus inhibiting the increase in adiposity associated with low levels of these hormones [138,139]. Adenovirus 36 (ad36), known to enhance the obesity risk in humans by causing inflammation and adiposity, is a possible target for prophylactic anti-obesity vaccination [140,141].

The discovery of the brite adipocyte type unveils it as a promising therapeutic target for obesity treatment. Induction of brown-like white adipose tissue adipocytes (beige cells) can counteract obesity-induced metabolic processes and increase weight loss through high levels of thermogenic gene expression [142,143]. Cold exposure is a promising non-pharmacological approach to shift the thermogenic program in beige adipocytes by activating β adrenergic receptor (ADRB) expression [144]. Among dietary compounds, capsaicin, found in red pepper, is the most studied browning activator [145,146]; however, several nutritional components are now known to play a role in browning induction. Together with capsaicin, cucumin [147], and n-3 Polyunsaturated fatty acids (PUFAs), particularly the eicosapentaenoic acid (EPA), found in fish oil, also activate beige cells by activating ADRB3. EPA [148], green tea catechins [149], and resveratrol [150] also function as epigenetic modulators, inducing activation of peroxisome proliferator-activated receptor γ (PPARy) and PRDM16 transcription factors. Resveratrol, EPA, curcumin, berberine [151], and all-trans retinoic acid [148] act directly in mitochondrial biogenesis by activating AMP-activated protein kinase (AMPK) pathway. Pharmacological activators of beige cells under studies include β3-adrenergic receptor agonist [152], PPARy and PPARα activators [153,154,155], PGC-1α stabilizer [156], and metformin as an AMPK activator [157,158].

The different obesity treatments existing or under study are summarized in Table 1.

#### 2.1.5. COVID-19 and Obesity

The recent pandemic of coronavirus disease, COVID-19, has been worsened by high levels of obesity and overweight in the world. The pathophysiological changes present in obesity, such as impaired immunity, chronic inflammation, and high blood pressure increase the risk of hospitalization in 113% and mortality by 48% in young individuals [159].

In obesity, abdominal fat compresses the diaphragm, restricting the airflow and decreasing lung capacity. Obstructive sleep apnea and other breathing disorders are common in obese individuals, which predisposes to hypoventilation-associated pneumonia, pulmonary hypertension, and cardiac stress. The large body mass also causes difficulties in intubation and mask ventilation [160].

Hormone and nutrient imbalance that are typical of obesity can impair adaptive and immune responses. Hyperglycemia can impair immune response, producing oxidants and glycation molecules [161]. Insulin and leptin signaling are crucial for T-cell activation, therefore, impairment of these pathways can lead to T-cell dysfunction [162,163]. Additionally, the chronic low-grade inflammation caused by constant high levels of leptin and other proinflammatory cytokines can decrease the immunity period covered by vaccines, as occurred with influenza vaccination.

## 3. Diabetes Mellitus

Diabetes mellitus is characterized by chronic hyperglycemia that impairs food metabolism. Causes of prolonged high levels of plasma glucose may be immune-mediated (type 1 diabetes), insulin resistance (type 2 diabetes), gestational diabetes, or others (neonatal, insipidus, brittle, LADA). Increased blood glucose leads to the classic diabetes symptoms: frequent urination (polyuria), increased thirst (polydipsia), and increased hunger (polyphagia), and can lead to the development of micro and macrovascular complications, resulting in nerves, heart, kidney, skin, and retina diseases [164]. Diabetes is a major global health issue. In 2019, about 463 million adults (from 20 to 79 years old) were living with diabetes and this number could increase to 700 million by 2045, causing 4.2 million deaths worldwide and being considered the fastest growing global health emergency [165].

Under normal metabolic conditions, food ingestion triggers insulin secretion by pancreatic β-cells, which induces glucose uptake in peripheral tissues and suppresses endogenous glucose production. Insulin acts directly on skeletal muscle, liver, and adipocytes via specific signaling pathways to induce various processes involved in glucose homeostasis [166]. In muscle, insulin improves glucose utilization by increasing the glucose transporter, GLUT4, and storage, promoting glycogen synthesis [167]. In the liver, the hormone activates glycogen synthesis and regulates lipogenic and gluconeogenic gene expression programs [168]. In adipocytes, it stimulates glucose uptake and lipogenesis, while decreasing lipolysis [169]. All of these integrated processes work simultaneously to keep blood glucose levels constant. To maintain the homeostasis, the blood glucose level must be sustained within a small interval despite the oscillations in supply and demand that occur in fasting/feeding cycles. Failures in insulin signaling block glucose uptake, leading to a prolonged hyperglycemic state [166].

Diabetes is characterized by β-cell failure, which can be auto-immune due to β-cell destruction, or by a progressive impairment of β-cells function that leads to insufficient insulin secretion. If insulin secretion is insufficient to regulate glucose uptake in peripheral tissues, β-cells need to increase the amount of secreted insulin in order to lower plasma glucose, a process called IR. The stress caused by constant overproduction of insulin can lead to β-cell failure followed by cell death [170].

### 3.1. Type 1 Diabetes Mellitus

In type 1 diabetes mellitus (T1D), insulin deficiency results from loss of pancreatic β-cells due to autoimmune-mediated destruction. This pathogenesis is a continuum disease that initiates with an early asymptomatic stage with auto-antibodies detection, this stage occurs years before the development of symptoms. Gradually, a decline in β-cell mass and dysglycemia that evolves to symptomatic T1D, which presents typical symptoms of hyperglycemia, such as weight loss, hyperphagia, and polyuria [171]. T1D is one of the most common metabolic diseases occurring in childhood, with more than 1.1 million children and adolescents affected in 2019 [165].

T1D is determined by genetic susceptibility, ineffective immune system, and environmental factors. A genome-wide association study and meta-analysis found 40 genetic loci associated with this disease [172]. Particularly, the HLA region on chromosome 6 has been identified as a T1D predisposition locus. This region provides half the susceptibility that leads to T1D risk; however, most loci associated with disease development are thought to involve immune responses, supporting the idea that genetic influences involve mechanisms that contribute to aberrant immune responsiveness [173].

The T1D autoimmune process begins with the activation of CD4+ T-lymphocytes, responsible for the secretion of IFNγ, macrophages and antigen presenting cells (APCs), such as dendritic cells (DCs). These cells generate antibodies to β-cell, which lead to chronic immunological responses, such as the secretion of cytokines (e.g., TNFα and IL-1) and activation of lymphocytes and NK cells. These activated cells work together to destroy pancreatic β-cells, inducing structural changes that suppress their ability to release insulin, leading to the development of T1D (127). This process produces various specific pancreatic islet auto-antibodies that are involved in the further development of the disease, including (I) islet cell autoantibodies (ICA), (II) glutamic acid decarboxylase auto-antibodies (GADA), (III) insulinoma associated 2 auto-antibodies (IA-2A), (IV) insulin auto-antibodies (IAA), and (V) recently described zinc transporter auto-antibodies (ZnT8A). These molecules are extremely important, mainly for patients with a non-canonical T1D phenotype, and are used to predict and confirm autoimmunity [174].

There is also the involvement of environmental factors in T1D development, such as viral infections, timing of the food introduction and gestational events. The contribution of exposure to these events on the development of T1D is believed to be small, but a combination of events can trigger the onset of a first β-cell auto-antibody [175].

### 3.2. Type 2 Diabetes Mellitus

Type 2 diabetes mellitus (T2D), also known as non-insulin dependent diabetes mellitus, is the most common form of diabetes, accounting for about 90% of all diabetes cases worldwide, according to the International Diabetes Federation [165]. This type of diabetes is characterized as an endocrine and metabolic disorder that associates environmental factors, such as energy-dense ‘Western’ nutrition, sedentary lifestyle, stress, aging, and obesity, with genetic factors, resulting in β-cell dysfunction and IR [176]. Although the genetic factor plays a significant role, the major cases of T2D are potentially preventable with a healthy diet and active lifestyle [177].

Prior to achieving the hyperglycemia that characterizes T2D, individuals manifest a stage of prediabetes. At this stage, the individual may present high fasting glucose levels, impaired glucose tolerance, and increased glycated HbA1c levels. Other biomarkers are high blood concentrations of proinflammatory cytokines, such as IL-6 and TNFα [178], gut microbiota profiles [179], and decreased sex hormone-binding globulin [180]. Prediabetes can be reversed through behavioral management, such as diet and sedentary lifestyle improvement. Increasing intake of whole grains and green leafy vegetables and lowering intake of highly processed and sugar-sweetened foods, and alcohol, combined with regular physical activity, can decrease the disease conversion to diabetes [177].

The causes, symptoms, and progression of this disease can vary substantially among individuals, but the main mechanism is the progressively impaired insulin secretion by pancreatic β-cells. IR decreases the efficiency of tissue glucose uptake by multiple abnormalities. The main tissues affected by IR are liver, muscle, and adipose tissue. However, this deficiency also affects pancreatic β-cells [181], intestinal metabolism [182], kidney [183], brain [184], and vasculature [185].

In the liver, additionally to IR, deficiency in insulin production and excessive production of glucagon (hyperglucagonemia) increase glucagon sensitivity and delivery of metabolic substrates, such as FAs, lactate, and glycerol. This leads to an increase in gluconeogenesis, despite the presence of fasting hyperinsulinemia and causes impaired suppression of insulin-responsive hepatic glucose production [186]. In muscle, IR affects glucose transport and phosphorylation, mitochondrial activity, glycogen synthesis, and pyruvate dehydrogenase complex activity [187]. The elevated glucose caused by dysfunctional uptake and gluconeogenesis lead to glucotoxicity in these tissues.

In adipose tissue, IR impairs the suppression of lipolysis and the release of FFA that normally occurs in high levels of insulin. Defective insulin signaling leads to glucose intolerance and triggers the efflux of FFA into circulation, thereby inducing a proinflammatory state [188]. Altered lipid metabolism can activate toll-like receptors (TLRs), affecting inflammation. In T2D, adipose tissue presents a high rate of macrophage infiltration and increased levels of proinflammatory cytokines and adipokines, such as leptin. These proinflammatory cytokines and high FFA levels can activate downstream kinases, such as TNF, IκB kinase-β (IKKβ), JUN amino-terminal kinase 1 (JNK1), and p38 MAPK, which induce phosphorylation on serine residues of the insulin receptor substrate (IRS) proteins. Moreover, these kinases may enhance the production of protein suppressors of cytokine signaling (SOCS) that block IRS action [189]. On the other hand, increased levels of IL-6 can stimulate hepatic gluconeogenesis, also inducing IR. Macrophage infiltration into adipose tissue increases proinflammatory M1 macrophages and T helper cells, while decreasing M2 macrophages and regulatory T cells, stimulating lipolysis itself [190].

T2D is associated with increased morbidity and mortality due to the development of complications that affect several organs. The life span of diabetic individuals is shortened by an average of 6 years, and the loss in life expectancy can reach 12 years in young onset development of T2D [191]. Diseases associated with T2D are divided in two categories: (I) macrovascular complications, such as CVD, that encompass coronary heart disease, peripheral vascular disease and cerebrovascular disease, which is a major motive of death and disability [192]; and (II) microvascular complications, due to severity and duration of hyperglycemia [193]. This includes retinopathy, neuropathy, and chronic kidney disease, which accounts for about 10% of deaths among diabetics [194]. The molecular mechanisms that contribute to the macro and microvascular complications are the same: reactive oxygen species (ROS) activate several proinflammatory pathways resulting in epigenetic changes. Thus, the expression of proinflammatory genes continues even after the normalization of glycemia [195].

### 3.3. Gestational Diabetes Mellitus

Gestational diabetes mellitus (GDM) occurs with the spontaneous development of hyperglycemia during pregnancy. Advanced maternal age, family history, poor eating habits, and obesity are the main risk factors for the development of this disease, which affects about 14% of pregnancies worldwide [165]. As pregnancy demands more energy, in early stages, there is an increase in insulin sensitivity, allowing greater glucose uptake in the adipose tissue. However, as pregnancy progresses, local and placental hormones such as estrogen, progesterone, leptin, cortisol, placental lactogen, and placental growth hormone, promote a state of IR [196]. GDM increases the risk of preterm birth and preeclampsia in children, which can result in overgrowth, since there is an increase in the placental transport of glucose, amino acids and FAs, stimulating the production of insulin and IGF-1. Moreover, this abnormal insulin production can cause pancreatic β-cell dysfunction and IR, even prenatally [197]. Usually, GDM resolves at the end of the gestation period. However, it can have lasting consequences, such as increased risk of development of T2D, a CVD in the mother and predisposition to obesity and T2D in children.

### 3.4. Maturity Onset Diabetes of the Young

This type of diabetes belongs to the subgroup defined as early diagnosis, typically before age of 25, and is not insulin dependent. It is characterized as an autosomal dominant disease with heterozygous mutations in various transcription factors that act in the development and maturation of pancreatic β-cells [198]. Despite its genetic origin, maturity onset diabetes of the young (MODY) is a heterogeneous disease, with different medical conditions and treatments associated with each subtype. To date, 14 different genetic mutations have been reported to be related to MODY, each of them corresponds to a MODY subtype. The six major MODY-causing genes encodes hepatocyte nuclear factor 4α (HNF4α), HNF1α, glucokinase (GCK), pancreatic and duodenal homeobox 1 (PDX1), HNF1β, and neurogenic differentiation 1 (NEUROD1) [199]. Due to its heterogeneity, early diagnosis based on next-generation sequencing has been essential to set individualized treatments, preventing long-term diabetes complications [200].

### 3.5. Other Types of Diabetes

There are several other types of diabetes, which occur less frequently in the population. Diabetes insipidus is characterized by the excretion of large volumes of dilute urine due to vasopressin deficiency, arginine vasopressin (AVP) resistance, or excessive water intake. It is mainly caused by a decrease in AVP secretion or action, which may be partial or complete, which can be acquired, or a genetic defect in the neurohypophysis [201]. Brittle diabetes occurs in a small group of patients with T1D, mainly women, with severe glycemic instability, poor metabolic control, and a compromised quality of life due to very common acute complications, hospital recoveries, and appearance of chronic problems [202]. Diabetes can also be developed due to diseases of the exocrine pancreas, such as acute pancreatitis [203] or cystic fibrosis [204]. Some hormones, such as GH, glucagon, and catecholamines, can antagonize insulin action. This mechanism can be exacerbated in tumors that produce excess of these hormones, inducing IR. In this case, diabetes may disappear or ameliorates with tumor removal [205]. Drug- or chemical-induced diabetes can arise over the use of compounds toxic to β-cells (160). In addition, certain infections, such as congenital rubella and cytomegalovirus, are also associated with autoimmune destruction of β-cells [206].

### 3.6. Management of Diabetes Mellitus

In diabetes, glycemic control is achieved by administration of antidiabetic medications that reverse the effects of its pathophysiological damaged insulin signaling. There are different classes of antidiabetic treatments and their choice varies according to several factors, such as the nature of diabetes, age, and the progression of the disease. Effective treatment requires multiple actions to circumvent the various pathophysiological defects. The strategy must be based on all the known pathogenic abnormalities and many individual factors called “ABCDE” of diabetes, that are: body weight, complications, duration, education and expense, and etiology [164]. Early diagnosis and implementation of therapeutic strategies are the most efficient to prevent progression of diabetes mellitus. Due to the lipotoxicity caused by obesity and physical inactivity, lifestyle interventions are a part of all intervention strategies, with or without drug treatment, depending on the factors mentioned above [207].

#### 3.6.1. Dual Therapies

Insulin

Insulin has been widely used in patients with diabetes. Therapy is based on the patient’s weight and typical doses range from 0.4 to 1.0 units/kg/day, depending on the glycemia (always self-monitored), meal size, and tissue glucose demand. There are several types of insulin, which are categorized from fast-acting to long-acting, from insulin analogues to human insulins, primarily based on how it works and how quickly it acts [208]. Long-acting insulin analogues are thought to result in fewer hypoglycemic episodes and are given 1–3 times a day according to the patient’s pharmacokinetic properties to control glucose levels between meals and fasting. Postprandial insulin treatments comprise fast-acting analogues or regular short-acting insulin, which are given before each meal and each time a correction of high blood glucose is required, occurring mainly 3 times a day [209].

Insulin can be administered by two routes: injection or infusion. The injection can be done with syringes, which are injected into the fat layer just under the skin, or with insulin pens, which can be reusable or disposable. As an infusion, it can occur via a vein in the hospital, with constant supervision by specialists, or via insulin pumps, which are computerized devices programmed to transport insulin under the skin, considered more durable [208]. In T2D, insulin therapy is usually used after failure of other treatment strategies to control blood glucose and requires larger doses than T1D treatment. Insulin therapy is often combined with other antidiabetic drugs, and the most common combinations are with metformin or thiazolidinediones (TZD), but the combination with GLP-1, sodium/glucose co-transporter 2 (SGLT2) are also effective in lowering HbA1c blood levels [210].

Metformin

The most commonly prescribed antidiabetic drug is metformin, a biguanide that lowers hyperglycemia by reducing hepatic glucose production, which leads to decreased HbA1c and fasting plasma glucose. The mechanism of action of metformin is still unclear, but it is known to be related to mitochondrial dysfunction by inhibiting mitochondrial glycerophosphate dehydrogenase, mitochondrial complex I, and activation of AMPK, and has no effect on pancreatic β-cells function. Metformin is usually combined with other drugs that increase insulin secretion, such as sulfonylureas. Metformin alone does not improve muscle insulin sensitivity, and HbA1c progressively increases after the initial decrease [164,211]. Other examples of biguanides are phenformin and buformin.

SGLT2 Inhibitors

SGLT2 is responsible for about 90% of glucose reabsorption. SGLT2 inhibitors, such as canagliflozin, dapagliflozin, and empagliflozin, are used to lower glucose blood levels by preventing renal reabsorption of glucose, thereby increasing its excretion. This increased glucose excretion via glycosuria reduces blood glucose, which ameliorates glucotoxicity, improving β-cell function, and increasing insulin sensitivity [212,213].

Targeting GLP-1

GLP-1 is a peptide produced by the GI system in response to food intake and stimulates glucose-dependent insulin secretion while inhibiting glucagon secretion. However, the GLP-1 half-life lasts only a few minutes. It can be targeted to the diabetes treatment in two different forms: (I) targeting the GLP-1 receptor itself, with an incretin mimetic with an extended half-life; or (II) targeting dipeptidyl peptidase 4 (DPP4) enzyme that acts by inactivating GLP-1. DPP4 inhibitors, such as sitagliptin, vildagliptin, saxagliptin, linagliptin, and alogliptin, can prolong the half-life of GLP-1, thus improving the glycemic control in T2D [214]. GLP-1 receptor agonists, such as exenatide, liraglutide, lixisenatide, and dulaglutide, promote insulin secretion from pancreatic β-cells, inhibiting inappropriate glucagon secretion by pancreatic α-cells, delaying gastric emptying and controlling appetite. Moreover, this molecule can reduce pancreatic β-cell apoptosis, stimulate their proliferation and improve their survival rate, with a concomitant reduction in body weight, which is a positive effect, since diabetes is directly related to obesity [215].

#### 3.6.2. T1D Therapies

Cyclosporin

To circumvent the autoimmune destruction of pancreatic β-cells, treatment with the immunosuppressive agent cyclosporin was the first immunotherapy tried. Cyclosporin is a calcineurin inhibitor that acts directly on T cells and was first tested in the 1980s in patients on insulin therapy for less than 2 months after diagnosis. A successful remission rate of diabetes throughout the treatment was observed; however when it was stopped, the disease progressed and resulted in the destruction of the residual β-cell mass, since the treatment could not be prolonged due to its effects, such as nephrotoxicity and an increased risk of cancer [216]. These results instigated researchers to investigate therapies that promote immune-tolerance, rather than immunosuppression, as well as short-term strategies to re-educate of these patient’s immune systems. Since then, various therapies have been tested, targeting T-cells, β-cells, antigen specific, among others, but many unanswered questions remain, especially regarding the mechanisms behind the development of this autoimmune disease [217].

Pramlintide

Pramlintide is administered adjunct with insulin treatment, which consists of injectable and oral glucose lowering drugs. Its active compound in the pramlintide acetate (SYMLIN) injection, is an amylin analogue and the first non-insulin T1D treatment. It reduces postprandial glucose concentrations, improves overall glycemic control and promotes a significant weight reduction [217]. Amylin is a 37 amino acid neurohormone co-secreted with insulin by pancreatic β-cells after a meal and its levels are reduced in T1D, and pramlintide is a synthetic analogue of amylin that was approved in April 2004 by the FDA [218].

Surgical Interventions

In some cases, pancreatic transplantation is an option for the patient with T1D. The procedure consists of a surgical operation with the normal pancreas of a decreased person inserted into the patient. After this procedure, the new pancreas produces insulin and this hormone therapy is not necessary anymore, but a special care is necessary with rejection of the new organ, being required the use of anti-rejection drugs or immunosuppressants for the rest of life. In addition to this, there is also pancreatic islet transplantation, being called allotransplantation, which consists of the purification and transfer of islets from a dead donor to the patient, resulting in the reestablishment of insulin secretion and is performed in patients with uncontrollable T1D levels [208]. However, both transplants carry the risk of sensitization against the same autoimmune antigen that led to prior β-cells collapse [219].

#### 3.6.3. T2D Therapies

Sulfonylureas and Glinides

Sulfonylureas are oral hypoglycemic medications that lower glucose levels in the blood plasma by increasing insulin secretion. The high insulin level overcomes IR and lowers the HbA1c levels in the blood. However, as with metformin action, sulfonylureas had no long-term effect on blood glucose and HbA1c levels increased progressively after the initial decline. They trigger insulin release by directly binding and closing the ATP-sensitive K^+^-channels of β-cell plasma membrane, which provokes membrane depolarization, opening the voltage-sensitive Ca^2+^ channels, leading to the release of mature insulin granules. Despite their wide use in diabetes treatment, sulfonylureas are known to be associated with hypoglycemia, weight gain, increased risk of cardiovascular events and may even accelerate β-cells failure [220]. Another class of anti-diabetics that have similar mechanisms of action are the glinides. Drugs such as repaglinide and nateglinide are short-action insulin secretagogues, which are highly effective in lowering HbA1c blood levels. However, due to the short-term action, they require administration before each meal [221].

Thiazolidinediones (TZDs)

TZDs, such as pioglitazone and rosiglitazone, are insulin-sensitizing drugs that enhance insulin sensitivity in skeletal and cardiac muscle, liver, and adipocytes. Their mechanism of action occurs through activation of PPARγ, a nuclear receptor that regulates the transcription of several genes involved in glucose and lipid metabolism and energy balance, such as GLUT4, glycogen synthase, and pyruvate dehydrogenase. PPARγ activation increases fat oxidation, proliferation of adipocytes, lipogenesis, fat redistribution, and adiponectin levels, and reduces plasma FFA levels and pro-inflammatory cytokines [222]. Despite their anti-diabetic effects, the use of TZDs presents several adverse effects, such as fluid retention, weight gain, and trauma-related fractures. Hence, compounds with similar anti-diabetic effects, but with attenuated secondary effects are targets in the search for new anti-diabetics [223].

Alpha Glucosidase Inhibitors (AGIs)

AGIs have no effect on insulin secretion or sensitivity, they slow the carbohydrate absorption by averting alpha-glucosidases from converting polysaccharide carbohydrates to monosaccharides in the GI system, thus lowering post prandial blood glucose levels. Some GI adverse effects, such as diarrhea, nausea, and abdominal pain, are related to the use of AGIs [224].

As a summary, current approaches to the diabetes treatment are shown in Table 2.

### 3.7. COVID-19 and Diabetes

Data recorded during 2019 in China (179) showed that patients with severe disease had a higher prevalence of diabetes (16.2%) compared to those with non-severe disease (5.7%), and COVID-patients with diabetes had higher mortality, 7.3% versus 2.3% overall [225]. However, it should be noted that diabetes has been associated with a poor prognosis in other viral infections. 

Many hypotheses are emerging to explain the relationship between diabetes and COVID-19. The first is that diabetic patients have an exaggerated proinflammatory response in the absence of appropriate immunostimulation by increasing the cytokines IL-1, IL-6, and TNFα, and this response could be more exaggerated with SARS-CoV-2 infection [226]. COVID-19 positive individuals with diabetes have been shown to have significantly increased levels of IL-6 and C-reactive protein compared to COVID-19 patients without diabetes [227]. Thus, diabetic patients may have a potential organ damage after SARS-CoV-2 infection by the exacerbate cytokine response, increasing mortality rates [228]. On the other hand, the overexpression of angiotensin-converting enzyme 2 (ACE2) in diabetic patients due to the use of ACE inhibitors (ACEi) or angiotensin-receptor blockers (ARBs), favors the entry of the virus in the host [226]. It should be noted that ACE2 is expressed in the pancreas, so the entry of SARS-CoV-2 into pancreatic islets may produce a β-cell dysfunction, and, consequently, a hyperglycemic state [229].

## 4. Metabolic Associated Fatty Liver Disease (MAFLD)

MAFLD, formerly known as non-alcoholic fatty liver disease (NAFLD), is a spectrum of diseases ranging from steatosis, characterized by an abnormal hepatic lipid accumulation; including non-alcoholic steatohepatitis (NASH), defined by liver inflammation and steatosis; that, when aggravated, it can lead to fibrosis, and, ultimately, evolve to cirrhosis and even hepatocellular carcinoma (HCC) [230]. MAFLD is estimated to affect about 1.8 billion people worldwide, comprising about 25% of the world’s population [231]. Although silent in many cases, MAFLD is the most prevalent liver disease among the population and its burden is expected to increase in the coming decades [232]. Despite these surprising facts, the MAFLD is still absent from global public health policies and there are still no approved pharmacological treatments for it.

The definition of MAFLD comprises the evidence of fat accumulation in the liver in addition to at least one of the following comorbidities: overweight/obesity, T2D, or evidence of metabolic dysregulation (e.g., larger waist circumference; elevated blood pressure, high TG or plasma HDL levels, prediabetes, homeostasis model assessment of insulin resistance (HOMA-IR) scores ≥2.5, and high plasma high-sensitivity C-reactive protein levels) [233].

Well-known hallmarks for the development and progression of MAFLD are IR, mitochondrial dysfunction, lipotoxicity, and inflammation. Dietary intake, such as excessive fructose and fat consumption, environmental factors, and genetic predispositions contribute to disease progression [230]. MAFLD is a multifactorial pathogenesis that evolves from abnormal TGs accumulation, comprising more than 5% of hepatocytes volume [234]. Several molecular dysregulations are associated with this event: elevated FA uptake, white adipose tissue (WAT) lipolysis, enhanced de novo lipogenesis (DNL), and defects in insulin signaling [235].

In MAFLD, the major source of lipids in hepatocyte accumulation is TG derived from WAT lipolysis (up to 60%), induced by irregularities in the insulin pathway. Impaired insulin signaling, caused by poor eating habits and by sedentary lifestyle, results in increased lipolysis in WAT [236]. This generates an influx of TG into the liver, leading to substrate overload and, consequently, the development of hepatic IR that leads to intensified accumulation of DNL and TG [237]. Under IR condition, the phosphodiesterase 3B enzyme (PDE-3B) is not active, inhibiting the protein kinase A (PKA) and the hormone sensitive lipase (HSL). Consequently, lipolysis is not suppressed, increasing levels of circulating FAs [238], which are harvested by CD36, FATP, FABP, and caveolin-1 transporters [239]. Once inside, these FAs are esterified to TGs by diglyceride acyltransferase (DGAT) 1/2, which are stored in lipid droplets or exported via VLDL, very low-density lipoprotein (LDL) (194). MAFLD patients have higher VLDL production, suggesting that even this greater export of TGs is not able to compensate for the increased uptake of FAs [240].

Hepatic DNL accounts for 25% of TG accumulation in MAFLD [236]. Excess fructose and impaired insulin signaling stimulate DNL through the action of different transcriptional factors, such as carbohydrate response element binding protein (ChREBP), PPARγ, and sterol response element binding protein 1c (SREBP-1c), which are responsible for increasing gene transcription of glycolysis, and DNL, such as hepatic pyruvate kinase in the former, and ACLY, FASN, and SCD1 in the latter [241].

Dietary TGs are transported into the circulation as chylomicrons [242] that are captured by the liver via LDL receptor (LDLR) and LDLR-related protein 1 (LRP1) [243]. After metabolizing, FA are exported from the liver packed in VLDL particles, accompanying cholesterol, phospholipids, and apolipoproteins [244]. In MAFLD, this mechanism is impaired by hepatic IR, which stimulates DNL without inhibiting VLDL production [245].

Fructose metabolism is another important pathway for hepatic lipid accumulation that stimulates hepatic DNL, which ultimately contributes to lipid accumulation in hepatocytes [246]. In healthy individuals, this pathway contributes up to 5% of total hepatic TGs; however, in individuals with MAFLD, this contribution can reach 23%. Thus, this considerable increase suggests that upregulation of fructose metabolism is associated with MAFLD progression [247].

One major hallmark that differentiates NASH from steatosis is the occurrence of hepatocyte damage, that is mainly associated with oxidative and endoplasmic reticulum (ER) stress caused by lipotoxicity and necroinflammation. Elevated levels of non-esterified fatty acids (NEFA) are extremely toxic to hepatic cells, a phenomenon called lipotoxicity [248], which increases hepatic gluconeogenesis [249]. The greater amount of acetyl-CoA, produced from FA oxidation, inhibits the pyruvate dehydrogenase complex (PDH), which redirects pyruvate to glucose production [190]. However, the malonyl-CoA produced in DNL initial stages inhibits carnitine palmitoyltransferase 1A (CPT1A), that, ultimately, leads to downregulation of mitochondrial oxidation [250].

Over time, excess FA causes mitochondrial stress, leading to mitochondrial uncoupling, ROS production, and JNK activation [251]. In addition, other lipids in hepatocytes, such as lysophosphatidylcholine, ceramides, cholesterols, and diglycerides, can trigger hepatic IR and cell death [248]. These lipids are associated with increased ER stress, oxidative damage, and activation of NLRP3 inflammasome, which can damage hepatocytes and cause cell death by apoptosis, pyroptosis, and necropoptosis [252].

In addition to dysregulation of hepatic metabolic pathways, excess NEFA together with pathogen-associated molecular patterns induce inflammation by activating TLRs [253]. TLRs activate the pro-inflammation transcription factor NF-kB in hepatocytes, Kupffer cells (KC) and hepatic stellate cells (HSC) [254,255]. During increased inflammation, there is also an increment in the activity of NADPH oxidase, an enzyme responsible for ROS production in KC, which contributes to increased oxidative stress [256]. Through the production of ROS, KCs stimulate inflammatory signaling, mainly through the chemoattraction of other leukocytes [257]. Meanwhile, infiltrated pro-inflammatory macrophages stimulate the inflammatory process, which contributes to a vicious cycle of inflammation. Increased inflammation is strongly related to hepatic IR. The signaling of NF-kB and JNK, through TNFR1, RANKL, and ILR receptors, promotes the action of IKKb, a protein associated with increased IR due to phosphorylation of IRS-1/2 in hepatocytes [258].

Excess FAs and Ca^2+^ also induce mitochondrial adaptations, increasing ROS production and oxidative stress [259,260]. Obesity, even without MAFLD, increases mitochondrial respiration to its maximum [261]. However, obese individuals with NASH show an approximately 40% decrease in maximal respiration compared to healthy individuals, which may be associated with hepatic IR, mitochondrial uncoupling, and leaking activity [251]. In this pathway, chronic excess of mitochondrial acetyl-CoA leads to increased ROS production, decreased antioxidant capacity, and ATP depletion [262,263]. Increased ROS production also leads to oxidation of mitochondrial DNA, depolarization of the membrane, and translocation of cardiolipins to the cytosol, inducing cellular death [264].

In addition to these two hepatic apoptosis pathways, adipose tissue also contributes to fibrogenesis in MAFLD, increasing the secretion of proinflammatory cytokines and unbalancing secretion of leptin and adiponectin [265]. Both KC and HSC cells respond to leptin [265,266,267]. In KC cells, leptin upregulates TGF-β, inducing activation of HSC cells. In HSC cells, leptin induces the matrix metalloproteinase-1 inhibitor, TIMP-1, and collagen 1 production, while repressing matrix metalloproteinase 1. Leptin also upregulates microRNA 21, inducing the profibrogenic TGF-β/Smad pathway. In addition, leptin also upregulates a hedgehog pathway that keeps the activated phenotype of HSCs [266]. After NASH establishment, the aggravation of necroinflammation and fibrogenesis, immune cell infiltration, and activation of hepatic progenitor cells contribute to the disease progression from NASH to cirrhosis, and even to HCC [235].

In addition to the aforementioned environmental and metabolic factors, there are also genetic factors associated with MAFLD development. The best-known mutations are in patatin-like phospholipase 3 (PNPLA3), a gene that encodes a lipase [268]. Individuals with PNPLA3 mutations have lower DNL and lower expression of SREBP-1c [269]; however, they have increased levels of hepatic TG and decreased secretion of VLDL [270]. This genetic mutation is more common in the Latino population than in any other ethnicity [271] and increases the risk of NASH and HCC [272]. The molecular mechanism associated with PNPLA3 mutation is still poorly understood, but it is known that HSC cells have high expression of these genes [273]. Therefore, PNPLA3 mutations may be associated with greater activation of these cells, which increases the inflammation and fibrinogenesis. In the last few years, two other genes have gained attention as genetic risks of MAFLD. They are missense variants at the TM6SF2 and GCK receptor (GCKR) loci, associated with the disease severity and progression. In particular, TM6SF2 is associated with an increased CVD risk by increasing circulating LDL-cholesterol, and GCKR mutation is associated with MODY individuals [274].

The high prevalence of childhood obesity also increased the incidence of pediatric MAFLD which, in turn, is associated with increased overall mortality compared to the general population [275,276]. Additionally, MAFLD is recognized as a risk factor for CVDs in obese adult population, but this relationship is still discussed in children [277,278,279]. Recently, MAFLD criteria were evaluated in obese children, finding that diagnosis based on more than one MAFLD criterion is more accurate in this selected population, providing better identification of individuals with higher cardiometabolic risk and prediabetes. Thus, revealing the lack of more accurate description of MAFLD criteria in the context of childhood obesity [280].

### 4.1. Management of MAFLD

Currently, there is no definitive pharmacotherapy to treat the MAFLD spectrum. However, due to their metabolic dysfunctions interrelated with obesity and T2D, several treatments for the latter diseases are interchangeable. As with all other metabolic diseases, effective lifestyle changes, such as healthier dietary and increased physical activity, are beneficial strategies. Overall weight loss can reduce levels of intrahepatocellular lipids that damage liver cells [281]. Moreover, daily exercise training, as well as high fiber and protein intake, combined with a shift in major calorie intake for the morning meal are beneficial in MAFLD treatment [282]. In addition, pharmacological treatment can be used as an adjunct to these lifestyle modifications as bariatric surgery in morbidly obese patients, driving to gradual weight loss over time [283].

### 4.2. Anti-Diabetic Drugs

IR is the central factor behind toxic fat accumulation in the liver, as well as in steatohepatitis and fibrosis progression [284]. Thus, therapies focused on IR are also efficient in MAFLD treatment. Insulin sensitizers are a group of antidiabetic medications that have been proven to be effective in MAFLD treatment. Metformin treatment demonstrated improvement in aminotransferase levels and IR in diabetic and non-diabetic patients. However, it did not affect liver histology such as steatosis, inflammation, ballooning hepatocellular injury, and fibrosis pattern [285].

Treatment with TZDs increases FFA uptake by adipose tissue, lowering fat deposition in the liver, increasing hepatic lipogenesis and insulin sensitivity. Moreover, TZDs can also upregulate adiponectin, an anti-steatogenic adipokine [286]. Pioglitazone treatment was also able to reduce inflammation and fibrosis. Nevertheless, the use of TZDs have been restricted due to its increased risk of CVD development, congestive heart failure, bladder cancer, and bone loss [283].

Another class of antidiabetic medication used to treat MAFDL are the GLP-1 analogues. Liraglutide or exenatide treatments increase pancreatic insulin release and stimulate β-cell growth [287]. Liraglutide treatment demonstrates promising results in delaying fibrosis progression [288]. DPP-4 inhibitors, molecules that target GLP-1 receptors, are also used in MAFLD treatment [289].

SGLT2 inhibitors, such as canagliflozin, dapagliflozin, and empagliflozin, are glucose-lowering agents that have cardiovascular and renal protective action. T2D patients who also have MAFLD treated with SGLT2 inhibitors presented reduced liver fat content and achieved better biological markers of MAFLD, such as serum liver enzymes [290].

Obeticholic acid (OCA) is a synthetic bile acid with improved affinity for the farnesoid X receptor (FXR) [291]. Activation of FXR results in improved glucose metabolism and insulin sensitivity [292], reduced lipogenesis, and enhanced β-oxidation [293]. Bile acids also presented anti-inflammatory [294] and antifibrotic action [295].

### 4.3. Antilipidemic Agents

Dyslipidemia drugs are another approach to MAFLD treatment. Statins have been demonstrated to decrease hepatic FFA, steatosis, hepatic fibrosis, and the expression of inflammatory markers TNF-α and IL-6 [296,297]. PUFA use was able to improve overall symptoms and decrease TG and alanine transaminase (ALT) levels [298]. Fenofibrate and niacin were also promising in MAFLD treatment, but their use is not recommended due to possible hepatotoxicity and increased mortality risk [299].

Ezetimibe is a dyslipidemic medicament that acts by inhibiting cholesterol absorption. In studies with MAFLD, it showed promising results, as in histological observations, it was able to improve NASH and steatosis profiles. It also improves MAFLD biomarkers, such as aminotransferase, alanine aminotransferase, gamma-glutamyl transpeptidase, and LDL-cholesterol levels [300,301].

The stearoyl-CoA desaturase (SCD) inhibitor, Aramchol, initially used in gallstone treatment, has been shown to improve hepatic lipid accumulation in animal experiments, as well as in humans [302,303].

Acetyl-CoA carboxylase (ACC) inhibitors act by reducing DNL and inducing FA oxidation, thus decreasing hepatic FA content [304]. GS0976 is an ACC inhibitor that is under investigation in MAFLD treatment, which has shown improvements in hepatic lipid content, and biomarkers of fibrosis and apoptosis after 12 weeks of treatment (NCT02856555) [305,306].

### 4.4. Antioxidant Agents

Vitamin E is an antioxidant molecule used in NASH treatment to counteract the oxidative stress that leads to hepatocellular injury and disease progression [307]. Studies have shown that vitamin E has no impact on hepatic fibrosis. However, it was able to decrease aminotransferases levels, improve inflammation, steatosis, ballooning, and steatohepatitis in NASH subjects [308,309]. Still, vitamin E treatment is controversial due to its association with all-cause mortality [310], and increased risk of prostate cancer [311].

N-acetylcysteine (NAC) is another antioxidant agent that acts by increasing glutathione in hepatocytes. This mechanism reduces the amount of reactive oxygen species, thus limiting hepatocellular injury progression [312]. Betaine are nutritional antioxidants, also used in fatty liver treatment due to its anti-inflammatory, cytoprotective, antiapoptotic, and anti-steatogenic action, thus increasing insulin sensitivity [313].

### 4.5. Others

Elafibranor is a PPAR-α/δ agonist that has shown promising results in NASH treatment, with anti-inflammatory and antifibrotic effects, improving insulin sensitivity and liver function [314,315]. Another study demonstrates that elafibranor was able to resolve NASH without effects on fibrosis [316].

Pentoxifylline is a methylxanthine by-product that has been demonstrated to improve ALT levels, as well as steatosis, inflammation, and fibrosis inhibition TNF-α [317].

Probiotic therapy is an alternative to treat dysbiosis changes that are observed in MAFLD. Studies indicate that gut microbiome improvement by probiotics may ameliorate hepatic histology, inflammation, and biochemical markers [318,319]. Gut microbiome is also a target of IMM-124E, a hyperimmune bovine colostrum, which acts by reducing liver exposure to LPS and gut bacterial byproducts. This increases GLP-1, adiponectin, and regulatory T-cells, thus improving glycemic control [320].

Table 3 shows the different compounds under study used for the MAFLD treatment.

### 4.6. COVID-19 and MAFLD

Underlying liver diseases such as MAFLD [321,322] may also increase the risk of hospitalization and severity of COVID-19 [323]. Recent publications suggest that hepatic pro-inflammatory profile and increased ROS, characteristic of fatty liver patients, may worsen COVID-19 infection by intensifying the virus-induced inflammation [324,325]. Moreover, patients with liver disease had longer viral shedding time and increased rates of liver failure [326].

Liver fibrosis is another risk factor for COVID-19 severity. One study correlated the fibrosis-4 (FIB-4) index and the NAFLD fibrosis score (NFS) with COVID-19 severity [327]. Accordingly, intermediate and high FIB-4 and NFS scores were correlated with a higher risk of severe COVID-19. Additionally, patients with FIB-4 rate greater than 2.67 had higher risk of developing severe COVID-19, even in the absence of metabolic comorbidities. This state induces the release of hepatic pro-inflammatory cytokines that may also contribute to the exacerbation of virus-induced cytokine “storm” during the immune response to infection [327]. In this state, overproduction of proinflammatory cytokines disturbs coagulation pathways, creating imbalanced procoagulant and anticoagulant rates, thereby increasing the predisposition to microthrombosis, disseminated intravascular coagulation and multiple organ failure [328,329].

## 5. Cardiovascular Diseases (CVDs)

CVDs are still the main cause of mortality and morbidity worldwide [330], which is increasing globally [331], along with cardiovascular risk factors, such as obesity [332], T2D [333] and metabolic syndrome (MetS) [334]. The underlying cause of almost all CVDs, such as coronary vascular disease, cerebrovascular disease, venous thromboembolism, and peripheral vascular disease, is commonly preclinical atherosclerosis, which ultimately leads to myocardial infarction, cardiac arrhythmia, and stroke [330]. Although CVDs studies have been traditionally focused on the clinical aspects of the disease, numerous studies have shifted their focus on the metabolic basis of such conditions [331].

Cardiovascular function is heavily dependent on ATP availability, consequently demanding a constant supply of nutrients, i.e., fats, glucose, lactate, and ketones, to be used as fuel by the myofibrils. Cardiac cells use a wide range of metabolic pathways to obtain ATP, which include glycolysis, β-oxidation, tricarboxylic acid cycle or Krebs cycle, and oxidative phosphorylation [335]. Still, cardiovascular function is altered by MetS-associated metabolic alterations.

These MetS-associated cardiovascular risk factors, especially obesity, IR, and atherogenic dyslipidemia, lead to a myriad of vascular and cardiac diseases [336], including coronary atherosclerosis and calcification [337], cardiac dysfunction, myocardial infarction, and heart failure [338]. However, it is not completely understood how these risk factors contribute to the development of such a spectrum of cardiovascular conditions.

Evidence associates obesity and IR with an increased risk of CVDs [339]. Thus, obesity is especially related to two cardiovascular conditions: heart failure (also known as obesity cardiomyopathy) and cardiac atherosclerosis [340]. The major physical consequence of obesity is developing atherosclerotic CVDs, which increases the risk through risk factors brought by obesity, such as hypercholesterolemia, hypertension, hyperglycemia, atherogenic dyslipidemia, IR, proinflammatory state, and prothrombotic state [341]. However, obesity leads to alterations in the hemodynamic phenotype such as increased left ventricular mass [342]. In addition to that, although not fully understood, underlying molecular mechanisms, such as myocardial Ca^2+^ handling, are also deregulated, which is caused by changes in the expression of SERCA2A and ryanodine receptors, responsible for calcium transportation and, ultimately, leading to myocellular dysfunction in obesity and MetS [343].

A consequence of obesity to the cardiovascular system is high level of circulating FFA that results from enhanced glucose use and decreased FA oxidation. Thus, the heart adapts to this unbalanced metabolic profile by favoring increased FA regarding other metabolic fuels. This change in cardiac substrate availability, resulting in loss of myocardium fuel flexibility, has been associated with impaired cardiac function [344]. Further, the high levels of FA impair β-cell function and contribute to IR, this latter being directly linked to hypertriglyceridemia and considered a driver to CVDs [336]. Furthermore, evidence also suggests overexpression of FA transporters in Zucker rats [345].

As already mentioned, IR is another threat factor linked to cardiac dysfunction, increasing the risk of heart failure and atherosclerosis, especially in diabetic individuals [346]. A meta-analysis study with 516,000 participants argues that IR is the single most important cause of coronary artery disease and its prevention would avoid about 42% of myocardial infarctions [347]. Under IR condition, the switch between FA oxidation and glycolysis becomes impaired, which makes FAs the only source of energy in the heart. Consequently, the heart increases lipid uptake and accumulation and, ultimately, inflicts lipotoxicity [348]. As the switch between substrates depends on their availability through CD36 (for FA) and GLUT4 (for glucose) transporters, regulation of FA and glucose uptake and modulation of these transporters are possible therapeutic targets [349].

Evidence correlates IR with several cardiovascular events, namely, hypertension, fatal and nonfatal myocardial infarction, and sudden death [350,351], mainly through the development of dyslipidemia [346]. Such abnormal lipoprotein profiles have been associated with vascular inflammation and endothelial dysfunction [352], resulting in several CVDs, especially atherosclerosis [352,353]. Although not fully understood, evidence suggests that small dense LDL (sdLDL) and TG-rich lipoproteins (remnant lipoproteins) are atherogenic. HDL, on the other hand, is antiatherogenic, being characterized by enhanced reverse cholesterol transport, anti-inflammatory properties, protective capacity against LDL modification, among others [341]. Additionally, hypertriglyceridemia has been shown to increase the incidence of CVDs by about 76% in women and about 32% in men [354]. Moreover, the major drivers of dyslipidemia-induced CVDs are related to alterations in lipoprotein metabolism and increased release of FFA, causing lipotoxicity on endothelial cells [348].

Another well-known metabolic dysfunction related to CVDs is mitochondrial dysfunction, which includes structural changes in mitochondria [355], usually through formation of a giant mitochondria by enlargement of the mitochondria or the fusion of adjacent organelles by the action of fusion proteins, i.e., mitofusins 1/2 [356]. Other alterations in mitochondria include changes in the cristae and intramitochondrial creatine kinase crystals [357]. These abnormalities play a major role in various metabolic aberrations, i.e., enhanced oxidative stress, lower ATP production and energy supply, increased cell apoptosis, dysregulation of autophagy and ER stress, that ultimately contributes to CVDs pathogenesis [355]. Accordingly, restoring these cellular perturbations may be a significant therapeutic target that goes beyond the energetic impairment [355,358].

Therefore, each covered risk factor contributes through its own alterations in cardiovascular metabolism, which may overlap. Hence, taken together, it has been shown that CVDs have a complex etiology, in which multiple risk factors and pathological mechanisms contribute to their development.

### 5.1. Management of CVDs

#### 5.1.1. Primary and Secondary Prevention Strategies

Prevention strategies for CVDs are required globally to reduce the risk of major cardiovascular events and, ultimately, related deaths [359,360]. There is consistent evidence to support adequate risk reduction strategies in individuals who have not developed any CVD (primary prevention) and in individuals with established diseases (secondary prevention) [359]. Thus, the modification risk factors related to pathogenesis of CVDs and implementation of therapeutic strategies have been able to mitigate major risk of cardiovascular events [360]. Based on this, prevention guidelines include several recommendations, e.g., healthy eating, regular physical activity, avoiding of tobacco and alcohol, and achieving a healthy weight. Further, drug-based prevention strategies are based on controlling blood pressure, cholesterol, hypercholesterolemia, and platelet aggregation to prevent major cardiovascular events [359,360,361].

Since T2D increases the risk of CVDs, glycemic control is an important target to prevent coronary-related diseases. Since then, sulphonylureas, metformin, and insulin have been used to control glycemia [362]. Still, SGLT2 inhibitor drugs, e.g., empagliflozin, have been administered to reduce the risk and mortality of CVDs during T2D treatment [213]. Moreover, recent studies have introduced new glucose-lowering drugs, such as semaglutide, liraglutide, and empagliflozin, which can decrease CVD incidence [213,363,364].

Blood pressure-lowering drugs are another prevention strategy, effective in preventing strokes, coronary heart disease, and heart failure [365,366]. Some antihypertensive agents, such as β-blockers, ACE inhibitors, angiotensin receptor blockers, calcium channel blockers and diuretics, prevent the aforementioned CVDs, affecting metabolism, inflammation and oxidative states [16]. Furthermore, statins have also been used to prevent stroke, coronary-related diseases, and sudden cardiac death by decreasing levels of cholesterol and lipoprotein in the blood. However, more recently, ezetimibe and antibodies that inhibit proprotein convertase subtilisin-kexin type 9 (PCSK9) have been employed to reduce LDL levels [367,368]. Nevertheless, acetylsalicylic acid (ASA) is recommended for all patients with atherosclerosis, patients after a stroke or atrial fibrillation, and patients with acute coronary syndrome (ACS). Still, patients with ACS were medicated with P2Y12 inhibitors, i.e., clopidogrel, ticagrelor, and prasugrel, along with ASA, to inhibit thromboxane A2 (TXA2) [369,370]. In addition to those, anticoagulants and vitamin K agonists have been shown to be effective against ACS recurrence [360]. Furthermore, combinations of hypertensive agents (lisinopril and atenolol or hydrochlorothiazide), ASA, and statin improved blood pressure and cholesterol concentrations [371].

#### 5.1.2. Potential Therapeutic Targets

Recently, new therapeutic strategies have been investigated to reduce and mitigate CVD events, such as agents to modulate glucose and lipid metabolisms, mitochondrial targets, RNA-based therapies, and the endocannabinoid system.

Glucose Metabolism

Potential therapies to modulate the glucose metabolism in patients with CVDs risk have been investigated, with GLP-1 receptor agonists, DPP-4 inhibitors, SGLT2 inhibitors, and AMPK activators.

GLP-1 is a peptide hormone that mainly stimulates insulin secretion in β-cells, and its receptor is endogenously expressed in myocardial tissue and vascular endothelium. Evidence has demonstrated the protective role of GLP-1 receptor agonists in the cardiovascular system, decreasing abdominal visceral fat and systolic blood pressure, and improving endothelial and myocardial function [372], which ultimately reduces non-fatal stroke and the incidence of stroke and myocardial infarction [363,373]. However, GLP-1 is immediately subject to rapid degradation by DPP-4 [372] and, therefore, administration of DPP-4 inhibitors may reduce risk of major CVDs events [374,375]. In addition to those, SGLT2 plays an important role in glucose reabsorption in the kidney, and its inhibition decreases the blood glucose concentration, improving insulin sensitivity, reducing glucose toxicity and blood pressure, and inducing nephroprotection [376]. A study has shown that empagliflozin, an SGLT2 inhibitor, together with lipid-lowering therapy and antihypertensive medications reduce the incidence of risk factors, hospitalization, and death [213].

AMPK, considered the master metabolic regulator, has also been investigated as a therapeutic target for obesity, diabetes, and CVDs. Activation of AMPK directly phosphorylates several downstream targets and effectors, which are related to lipid metabolism (FA oxidation and DNL), glucose metabolism (glycolysis and glucose uptake), and mitochondrial integrity [377,378]. Furthermore, evidence suggests that AMPK activation yields cardioprotection, e.g., protects against hypertrophic cardiomyocyte growth and cardiac ischemia reperfusion injury [379,380]. As a result, many therapeutic agents currently used to treat diabetes can activate AMPK, including metformin and TZDs. The former has been shown to normalize the endothelial response via improved AMPK-induced nitric oxide (NO) production in animal models [381,382] and further analysis has demonstrated that metformin-induced AMPK activation suppresses 26S mediated GTP-cyclohydrolase degradation, which is the main mediator of endothelial dysfunction [383]. For the latter, there is evidence to support its cardioprotective properties, e.g., reducing ischemia, reperfusion, and myocardial infarction in mouse models [384].

Lipid metabolism

Elevated plasma lipoprotein(a) (Lp(a)) concentration has been associated with increased risk of CVDs [385]. Well-known agents, such as inhibitors of proprotein convertase subtilisin/kexin type 9 (PCSK9), nicotinic acid (niacin), statins and ASA, and novel molecules, such as antisense oligonucleotides (ASOs) and inhibitors of lipoprotein lipase (LPL) and its receptors, modulate Lp(a) levels. However, in clinical practice, no medication directly lowers Lp(a) levels, so the primary goal with patients with elevated Lp(a) levels is to reduce LDL-C levels [386].

Niacin administration studies have suggested that it reduces Lp(a), LDL-C, apolipoprotein (apo) B-100, sdLDL, and TG levels and raises HDL levels; however, clinical trials have reported that the role of niacin in lowering CVDs risk is questionable [387,388,389]. Furthermore, evidence indicates that ASA decreases serum Lp(a) concentrations, possibly achieved by reducing apo(a) gene transcription, which causes a reduction in LPA gene transcription [390]. Recently, PCSK9 inhibitors, e.g., evolocumab and alirocumab, have been combined with statins to reduce LDL-C, but this new class of medication has not yet been approved for the treatment of elevated Lp(a) levels [386,391]. However, the PCSK9-induced mechanism that lowers Lp(a) levels is unclear; hence, it has been hypothesized that a reduction in LDL-C and LDL-R may be involved in the lowering of Lp(a) levels [391].

Novel therapies have been evaluated to modulate Lp(a) synthesis. ASOs targeting apo(a) have been demonstrated to inhibit apo(a) synthesis and, consequently, Lp(a) secretion [392,393]. However, administration of an apo(a)-specific ASO, IONIS-APO(a)LRx, has been indicated to reduce plasma LDL-C levels and monocytes inflammatory effects, and, ultimately, Lp(a) levels [394]. Angiopoietin-like proteins (ANGPTLs), LPL inhibitors that hydrolyze circulating TG to FFA, have also been evaluated [395]. ANGPTL3 suppresses LPL activity, which reduces plasma levels of TG and LDL-C, while ANGPTL4 reduces plasma TG and increases HDL-C levels [395,396,397]. Both also play a role in glucose homeostasis [398,399,400]. Dewey et al. [401] showed that ASOs and monoclonal antibody-based inactivation of ANGPTL3 reduce plasma TG and LDL-C levels. In addition, a study demonstrated that metformin inhibits ANGPTL3 expression in the liver, modulating LPL activity and lowering plasma lipids [402]. Furthermore, LRP6 impairment exhibits elevated LDL, TG and fasting glucose levels, which also deregulate Wnt/β-catenin signaling and lipoprotein endocytosis [403]. With that, targeting LRP6 with small molecules, such as GNF-6231 inhibits the canonical and non-canonical effects of the Wnt ligand, slowing the progression of myocardial fibrosis and inflammation [404]. Another LRP6 antagonist is the Dickkof-related protein 1 (DKK1), which is an endogenous inhibitor that regulates blood pressure [405].

Mitochondrial Therapies

Mitochondrial dysfunctions play an important role in CVDs pathogenesis [355]; however, there are no medications to modulate mitochondrial functions available in clinical practice [406]. However, therapeutic agents have been investigated to target mitochondrial, including the mitochondria-targeted antioxidant MitoQ1, that decreases ROS production and has shown protective effect in hypertensive rat models [407], and carvedilol or antidiabetic drugs that prevent cardiac mitochondrial oxidative damage [408]. Therapies targeting endothelial NO synthase (eNOS) activity and function, using statins, ACE inhibitors, and AT1-receptor blockers, have been shown to improve cardiovascular prognosis by decreasing the level of oxidative stress, inflammation, and mediators of vascular dysfunctions [409,410]. In addition, third-generation β-blockers stimulate endothelial NO formation and improve oxidative stress in animal models [411,412].

#### 5.1.3. Emerging Strategies

RNA-Based Therapies

Over the past decades, RNA-based therapies have emerged as an important strategy for the diagnosis and treatment of many diseases [413]. However, insights into the role of non-coding RNAs (ncRNAs) in health and disease have characterized them as valuable therapeutic targets [414]. Although ncRNAs do not encode a protein, ncRNAs play an important role in several pathways through post-transcriptional regulation of gene expression [413]; thus, affecting different biological processes, such as cell survival, differentiation, and proliferation [415]. Due to their broad influence on biological processes, the role of ncRNAs, particularly microRNAs (miRNAs), have been explored in CVDs development [415,416,417,418], as well as their potential therapeutic applications [413,419,420,421].

Several studies have explored miRNA-based treatment in CVDs, such as myocardial infarction, cardiac fibrosis, and atherosclerosis. Upregulation of miR-146a in a myocardial ischemia/reperfusion injury in mice showed a 55% reduction in myocardial infarct size and an improvement in cardiac function after myocardial infarction [422]. Likewise, overexpression of miR-99a in C57/BL6 mice subjected to myocardial infarction attenuated cardiac remodeling by preventing cardiomyocyte apoptosis and promoting autophagy, cardiac function gain and increasing survival ratio [423]. Conversely, downregulation of miR-433 in mice ameliorates cardiac fibrosis and ventricular dysfunction after myocardial infarction [424]. Similarly, the administration of miRNA mimetics is also explored for CVDs treatment, which act on mRNA degradation and translation inhibition [425]. Thereby, systemic administration of a miR-100 mimic in an LDLR-deficient atherosclerotic mouse model decreased 55% of the plaque area, attenuating atherosclerosis [426]. In addition, intracardiac administration of miR-199a-3p and miR-590-3p mimetics immediately after myocardial infarction in mice led to cardiac repair, reducing infarct size and preserving cardiac function [427].

Since the discovery of miRNA in *Caenorhabditis elegans*, a deeper understanding of miRNAs functionality is still needed to translate it into clinical practice. With that, many ncRNAs have not yet been characterized, leaving a broad horizon of potential targets for the development of RNA-based treatments for CVDs with improved efficacy. Therefore, RNA-based therapies are a promising field of research for the treatment of several diseases, including CVDs, but their application remains a challenge for the scientific community.

Endocannabinoids

There is mounting evidence that the endocannabinoid system (ECS) influences the regulation of CVDs risk factors, such as hypertension and atherosclerosis [428]. Studies have explored ECS modulation, such as through endocannabinoids (e.g., anandamide (AEA), 2-arachidonoylglycerol), cannabinoid receptors antagonists (e.g., AM251), synthetic cannabinoids (e.g., WIN55212-2), or even related pathways (e.g., FA amide hydrolase inhibitors), to promote hypotensive effects in different types of hypertension, e.g., spontaneous, acute, and salt-induced hypertensions [429,430,431,432,433,434]. This evidence suggests that ECS-mediated hypotensive effects depend on the endocannabinoid or receptor involved and the type of hypertension being treated. However, further investigations are still required to elucidate the mechanisms behind the hypotensive effect and what are the appropriate therapeutic targets to be explored in clinical practice.

NO is an important cardiovascular signaling molecule [435]. In addition to playing a significant role in cardiovascular homeostasis [435], atherosclerosis [436], and renal damage [437], NO is also deeply involved in endocannabinoid-induced cardiovascular effects. AEA treatment promoted a notable relaxation of the thoracic aortas dependent on CB1 and CB2 activations [438]; however, when eNOS inhibitor, L-NAME, was administered, no AEA-evoked relaxation was observed, indicating that the AEA-induced vasodilation effect is also NO-dependent. Similarly, AEA-induced vasorelaxation was also reversed by L-NAME treatment in human mesenteric [439] and pulmonary [440] arteries, highlighting the role of NO in ECS-mediated vasodilation. Together, this evidence supports a central role for NO in cardiovascular homeostasis, as well as highlighting the NO contribution to the beneficial vascular effects induced by ECS.

Despite investigation of ECS strategies in hypertension treatment, the ECS have been shown to also play a role in CVDs development, especially by endothelial damage [441]. Endothelial CB1 signaling has been associated with proatherosclerotic effects by increasing oxidative stress and promoting immune cell recruitment into the arterial wall in atherosclerotic mice, apoE (−/−) [442]. Interestingly, a different study, also conducted with apoE (−/−) mice, presented that CB2 signaling promotes anti-inflammatory and anti-atherosclerotic effects [443]. Similar studies have shown that CB1 receptor activation in human primary coronary artery endothelial cells promoted cell-death and increased ROS levels [444]. However, CB1 activation promotes antioxidant effects in the digestive system [445] and CNS [446], highlighting that CB1 and CB2 receptors may exert different functions depending on their location.

Therefore, ECS is an exciting target for novel therapeutic strategies in CVD, especially those associated with hypertension and atherosclerosis. The broad distribution of the ECS throughout the organism makes it a versatile tool for targeting different diseases, given its wide range of modulation possibilities, such as receptor agonists and antagonists, metabolites, and enzymatic inhibitors and activators. However, CB1 and CB2 receptors can evoke opposite effects depending on their localization, making pharmacological vectorization strategies, such as nanotechnology, extremely important for accurate drug delivery.

The different compounds under study used to the CVDs treatment are shown as a summary in Table 4.

### 5.2. COVID-19 and CVDs

ACE2 is located in the cell membrane or circulating in the bloodstream. This enzyme is responsible for transforming angiotensin I into II, which is a potent vasoconstrictor agent. Thus, ACE2 is a blood pressure modulator. The SARS-CoV-2 entry into the host cell through the binding of the viral S protein to ACE2. Therefore, the interaction between S protein and ACE2 has been considered a promising therapeutic target [447]. CVDs and their risk factors, such as hypertension, were common pre-existing conditions in COVID-19 patients, with a prevalence of 15% of hypertension and 15% for other CVDs [448].

The use of ACEi and angiotensin-receptor blocker (ARB) therapy is a standard practice in hypertension treatment as we have discussed in the management of CVDs section. Recently, it has been questioned whether the use of antihypertensive medications would have a favorable or deleterious impact on people infected with SARS-CoV-2, since they can modulate ACE2 expression. Thus, possibly turning the users of ACEis and ARBs into susceptible individuals to increased entry and propagation of the viral host cells [449]. On the other hand, the treatment of COVID-19 patients with ACEi or ARB is not harmful [450,451], and the ACE2 modulation may be beneficial in patients with lung injury because of its anti-inflammatory effects [449,452].

## 6. Conclusions

**Figure 1:**
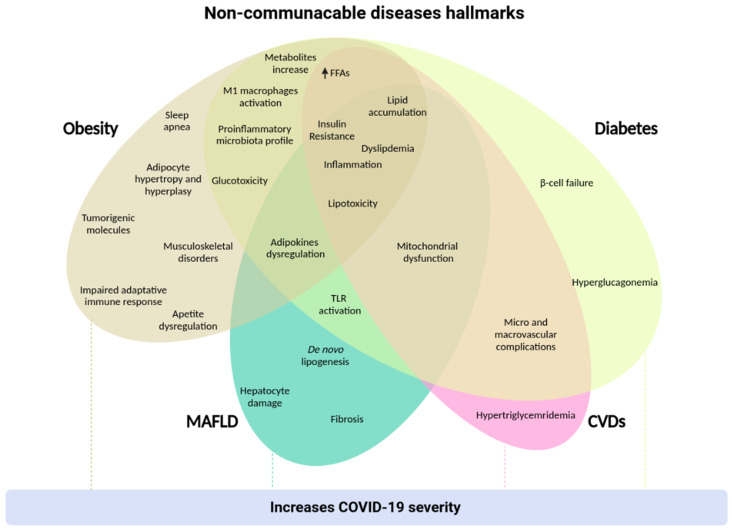
Hallmarks of non-communicable diseases. Scheme with the main characteristics and complications of the NCDs represented in 5-way Veen diagram and their relationship with COVID-19. Obesity is shown in the brown, metabolic associated fatty liver disease—MAFLD—in the blue, cardiovascular diseases—CVDs—in the pink, and diabetes mellitus in the yellow. The relationship between the diseases are shown in the intersections.

Taking all these metabolic alterations together, we observe that human metabolism is a finely tuned network of biomolecular interactions, from simple alterations, such as leptin and insulin resistance, to complex ones, such as modifications in metabolic enzymes and redox metabolism. These deregulations can lead to a wide range of diseases, including hormonal disorders, obesity, diabetes, MAFLD, CVDs, and cancer. With each one of these diseases having its own characteristics and complications, but of common origin that consists of an imbalance in the human metabolism (Figure 1). Each metabolism-related disease has its specific target tissues, e.g., liver cells in MAFLD, adipose tissues in obesity and diabetes, and a set of disruptions in the finely tuned metabolic network that characterizes it. However, all these alterations mainly came from the 21th century way of life, with high calorie diet, with sugary and fatty foods, and low physical activity. These factors are directly responsible for the increase in such diseases in recent decades; however, we must not ignore the genetic and epigenetic contribution to the development and progression of such diseases.

Treatments for these NCDs should integrate lifestyle changes and pharmacological or surgical approaches, when necessary, to successfully cure them or at least mitigate their burdens. Comprehensive lifestyle interventions, such as adequate physical training, psychological assistance, and dietary re-education, are the core step to combat the metabolic conditions covered in this review. The major barriers to successful lifestyle changes are long-term adherence to the proposed interventions and complete reeducation of patients’ lifestyle towards a more conscious one. Hence, in some situations, individuals fail to achieve the required weight loss or have compromised health; thus, adjunctive treatments, i.e., pharmacological or surgical interventions, may be required. Interestingly, pharmacological treatments tend to share targets and medications among NCDs, mainly due to shared or complementary metabolic changes. Thus, we showed some medications that are employed to treat more than one NCD, such as metformin, liraglutide, empagliflozin, and even treat more than one simultaneously. Therefore, treatments should be designed in an individualized manner to address the patient’s condition.

With this silent pandemic of NCDs that we are currently facing globally, its burdens are pronounced with the COVID-19. The clearest relationship between both pandemics are the increased risk of hospitalization, severity, and mortality, sharing molecular mechanisms, mainly related to inflammation and cytokine storm. However, further investigation is still required by the scientific community to fully understand the underlying relationship between these pandemics.

Therefore, reducing the burden of such metabolism-related diseases demands multidisciplinary approaches, which combine individual interventions with environmental and social changes. A better comprehension of notable regional specificities that contribute to the prevalence and trends of such diseases can help identify environmental and social causes and provide guidance on developing intervention strategies. In general, our comprehension of metabolism has become increasingly thorough in the past few decades. With these advances, our knowledge of underlying the mechanisms, progression and prognosis of diseases related to metabolic alterations are also deepening. Despite advances in recent years, more extensive research is still required to further improve diagnosis, therapy, and minimize the chance of chronic complications development. Additionally, accessing and reducing cost for high quality and powered genetic techniques will provide a wealth of information and opportunities for enhanced targeted treatment. Therefore, continuous investment in this field of research is essential to effectively target and mitigate the global pandemic of metabolic diseases-related we face.

## Figures and Tables

**Table 1:** Pharmacological treatments for obesity. The compounds are divided into class, mechanism of action, drugs examples, current state of application, and its side effects.

Class	Mecanism of Action	Drugs	Current State	Side Effects	Reference
Lipase inhibitors	Inhibit long-chain FAs absorption	Orlistat	Commercial	Abdominal pain Fecal urgency Flatulence Oil stool	[73,99]
	Induce weight loss Improve glycemic control Improve lipid profile	Cetilistat	Under studies	Diarrhea Flatulence Oily spotting	[136]
Adrenergic agonists	Increase norepinephrine release in CNS	Phentermine	Commercial	Increase heart rate and blood pressure	[99,100,101,102]
GABA receptor modulators	Neuroestabilizer Enhance thermogenesis	Topiramate	Commercial	Alteration of taste GI upset Nausea	[103,104]
POMC neurons activators	Induce α-MSH release	Naltrexone/Bupropion	Commercial	Nausea	[107,108,109,110,111,112,113]
	Delay gastric emptying Enhance insulin secretin	Liraglutide *	Commercial	Transient nausea Vomiting	[114,115]
GLP-1 receptor agonists	Reduce blood glucose levels Induce weight loss	Semaglutide TTP-054 ZYOGI	Commercial	Nausea Vomiting Diarrhea	[124,125,126,127]
	Induce insulin release Decrease HbA1c levels	ZP4165	Under studies	Unknown	[128]
Leptin analogues	Lower hepatic steatosis Improve insulin sensitivity	Metreleptin	Commercial	Nausea	[119,120,121]
Amylin analogues	Delay gastric emptying	Pramlintide * Davalintide *	Commercial	Nausea	[122,123]
Glucagon and GLP-1 receptors agonists	Supresses appetite Increase energy expenditure	Oxyntomodulin	Under studies	Unknown	[129]
GLP-1, glucagon and GIP receptors agonists	Induce weight loss	Triagonist 1706	Under studies	Unknown	[132]
CB1 antagonists	Stimulate anorexigenic signaling	AM-6545	Under studies	Unknown	[134,135]
Vaccines	Restrain appetite-stimulating hormones Decrease nutrient absorption	Anti-ghrelin Anti-somastatin Anti-ad36	Under studies	Unknown	[137,138,139,140,141]
Induction of beige-cells	Increase thermogenic gene expression Epigenetic modulators Activation of AMPK pathway	Capsaicin Curcumin PUFAs	Under studies	Unknown	[152,153,154,155,156,157,158]

* Similar compounds for more than one NCD.

**Table 2:** Pharmacological treatments for diabetes mellitus. The compounds are divided into class, mechanism of action, drugs examples, current state of application and its side effects.

Class	Mecanism of Action	Drugs	Type	Current State	Side Effects	Reference
Hormone	Reduce blood glucose levels	Insulin	1 and 2	Commercial	Hypoglicemia	[208,209]
Biguanides	Reduce hepatic glucose production	Metformin * Phenformin * Buformin *	1 and 2	Commercial	Abdominal discomfort	[169,211]
SGLT2 inhibitors	Prevent glucose reabsorption	Canagliflozin * Dapagliflozin * Empagliflozin *	1 and 2	Commercial	Urinary tract and genital infections Decrease in blood pressure Weight gain	[193,212,213]
DPP4 inhibitors	Improve glycemic control	Sitagliptin * Vildagliptin * Saxagliptin * Linagliptin * Alogliptin *	1 and 2	Commercial	Hypoglicemia Loss of consciousness Gastrointestinal side effects	[214]
GLP-1 receptor agonist	Promote insulin secretion	Exenatide * Liraglutide * Lixisenatide * Dulaglutide *	1 and 2	Commercial	Transient nausea Vomiting	[215]
Calcineurin inhibitor	Inhibit T cell activation	Cyclosporin	1	Commercial	Nephrotoxicity Increase risk of cancer	[216,217]
Amylin analogues	Reduce blood glucose levels Induce weight loss	Pramlintide *	1	Commercial	Nausea	[217,219]
Sulfonylureas and glinides	Increase insulin secretion	Tolbutamide Glibenclamide Glipizide	2	Commercial	Hypoglicemia Weight gain	[220,221]
PPARγ agonists	Increase tissues sensibility to insulin action	Rosiglitazone * Pioglitazone *	2	Commercial	Fluid retention Weight gain Trauma-related fractures	[222,223]
Alpha glucosidase inhibitors	Slow the carbohydrate absorption	Acarbose	2	Commercial	Diarrhea Nausea Abdominal pain	[224]

* Similar compounds for more than one NCD.

**Table 3:** Pharmacological treatments for metabolic associated fatty liver disease. The compounds are divided into class, mechanism of action, drugs examples, current state of application, and its side effects.

Class	Mecanism of Action	Drugs	Current State	Side Effects	Reference
Biguanides	Reduce aminotransferase levels Increase insulin sensitivity	Metformin *	Under studies	Abdominal discomfort Diarrhea Nausea	[285]
PPARy agonist	Increase FFA uptake, hepatic lipogenesis and insulin sensitivity	Rosiglitazone * Pioglitazone *	Under studies	Increase risk of CVD development, congestive heart failure, bladder cancer and bone loss	[283,286]
GLP-1 receptor agonists	Delay fibrosis progression	Liraglutide * Exenatide *	Under studies	Transient nausea Vomiting	[287,288,289]
SGLT2 inhibitors	Reduce fatty liver content Improve levels of serum liver enzymes	Canagliflozin * Dapagliflozin * Empagliflozin *	Under studies	Urinary tract and genital infections Decrease in blood pressure Weight gain	[290]
FXR receptor agonist	Regulate hepatic metabolism of bile and cholesterol	Obeticholic acid	Under studies	Pruritus Dyslipidemia Fatigue Headache Gastrointestinal side effect	[291,292,293,294,295]
Statins	Decrease hepatic FFA, steatosis, hepatic fibrosis and inflammatory markers	Atorvastatin Fluvastatin Lovastatin Pitavastatin Pravastatin Rosuvastatin	Under studies	Muscle pain (myalgia) Creatine phosphokinase elevation	[296,297]
Polyunsaturated fatty acids	Improve overall symptoms Decrease TG and alanine transaminase levels	Eicosapentaenoic acid Docosahexaenoic acid	Under studies	Unknown	[298]
Lipid lowering	Inhibit cholesterol and phytosterol absorption	Ezetimibe	Under studies	Hepatotoxicity Severe cholestatic hepatitis Acute autoimmune hepatitis	[300,301]
Stearoyl-CoA desaturase inhibitors	Improve hepatic lipid accumulation	Aramchol	Under studies	Unknown	[302,303]
Acetyl-CoA carboxylase inhibitors	Reduce DNL and FA content	GS0976	Under studies	Unknown	[305,306]
Antioxidants	Decrease aminotransferases levels Improve inflammation, steatosis, ballooning and steatohepatitis	Vitamin E	Under studies	Increase blood pressure and heart failure risk	[307,308,309,310,311]
	Increase glutathione in hepatocytes	N-acetylcysteine	Under studies	Nausea Vomiting Diarrhea Transient skin rash Flushing Epigastric pain Constipation	[312]
	Induce anti-inflammatory, cytoprotective, antiapoptotic and anti-steatogenic actions	Betaine	Under studies	Gastrointestinal side effects	[313]
PPAR-α/δ agonists	Induce anti-inflammatory and antifibrotic effects Improve insulin sensitivity and hepatic function	Elafibranor	Under studies	Congestive heart failure Peripheral edema Bone fractures Weight gain	[314,315,316]
Xanthine derivatives	Improve ALT levels, steatosis, inflammation and fibrosis	Pentoxifylline	Under studies	Unknown	[317]
Probiotic therapy	Improve hepatic histology, inflammation and biochemical markers	-	Under studies	Unknown	[318,319]
Hyperimmune bovine colostrum	Improve glycemic control Reduce liver exposure to LPS and gut bacterial bioproducts	IMM-124E	Under studies	Unknown	[320]

* Similar compounds for more than one NCD.

**Table 4:** Pharmacological treatments for cardiovascular diseases. The compounds are divided into class, mechanism of action, drugs examples, current state of application, and its side effects.

Class	Mecanism of Action	Drugs	Current State	Side Effects	Reference
GLP-1 receptor agonists	Reduce abdominal visceral fat and systolic blood pressure Improve endothelial and myocardial function	Liraglutide * Exenatide *	Under studies	Transient nausea Vomiting	[363,372,373]
DPP4 inhibitors	Degrade GLP-1	Sitagliptin * Vildagliptin * Saxagliptin * Linagliptin * Alogliptin *	Under studies	Hypoglicemia Loss of consciousness Gastrointestinal side effects	[374,375]
SGLT2 inhibitors	Promote glucose reabsorption Decrease blood glucose concentrationImprove insulin sensitivity Reduce glucose toxicity and blood pressure Induce nephroprotection	Empagliflozin *	Under studies	Urinary tract and genital infections Decrease in blood pressure Weight gain	[213,376]
AMPK activators	Induce NO production Suppress 26S mediated GTP-cyclohydrolase degradation	Metformin *	Under studies	Abdominal discomfort Diarrhea Nausea	[381,382,383]
	Reduce isquemia, reperfusion and myocardial infarction	TZDs	Under studies	Increase risk of CVD development, congestive heart failure, bladder cancer and bone loss	[384]
Lp(a)and LPL-C modulators	Reduce Lp(a), LDL-c, apo B-100, sdLDL and TG levels Raise HDL levels	Niacin	Under studies	Hepatic toxicity Myopathy Blurred vision Nausea Vomiting	[387,388,389]
	Decrease Lp(a) levels	ASA	Under studies	GI upset Nausea	[390]
	Reduce LDL-c levels	Evolocumab Alirocumab	Under studies	Nasopharyngitis Injection site pain Arthralgia Back pain	[386,391]
	Inhibit apo(a) synthesis and LP(a) secretion	ASOs	Under studies	Unknown	[392,393]
LPL inhibitors	Suppress LPL activityReduce TG level s Increase HDL-C levels	ANGPTLs	Under studies	Unknown	[395,396,397,398,399,400,401,402]
Mitochondrial therapies	Decrease ROS production	MitoQ1	Under studies	Unknown	[407]
	Reduce oxidative stress and inflammation	Statins ACE inhibitors AT-1 receptor blocker	Under studies	Unknown	[409,410,411,412]
RNA-based therapies	Reduce myocardial infarct size Improve cardiac function	miR-146a	Under studies	Unknown	[422]
	Prevent cardiomyocyte apoptosis Promote autophagy	miR-99a	Under studies	Unknown	[423]
	Ameliorate cardiac fibrosis and ventricular dysfunction	miR-433	Under studies	Unknown	[424]
	Attenuate atherosclerosis	miR-100	Under studies	Unknown	[426]
	Promote cardiac repair Reduce infarct size Preserve cardiac function	miR-199a-3p miR-590-3p	Under studies	Unknown	[427]
Endocannabinoids	Induce hypotensive and vascular effects	Anandamide 2-arachidonoylglycerol	Under studies	Neuropsychiatric side effects	[429,430,431,432,433,434]

* Similar compounds for more than one NCD.
